# A real-world prospective study on dialysis-requiring acute kidney injury

**DOI:** 10.1371/journal.pone.0267712

**Published:** 2022-05-05

**Authors:** Conrado Lysandro R. Gomes, Thais Lira Cleto Yamane, Frederico Ruzany, José Hermógenes Rocco Suassuna

**Affiliations:** 1 Clinical and Academic Unit of Nephrology, Hospital Universitário Pedro Ernesto, Faculty of Medical Sciences, Rio de Janeiro State University, Rio de Janeiro, Brazil; 2 Kidney Assistance LLC., Rio de Janeiro, Brazil; University of Sao Paulo Medical School, BRAZIL

## Abstract

**Background:**

Current information about acute kidney injury (AKI) epidemiology in developing nations derives mainly from isolated centers, with few quality multicentric epidemiological studies. Our objective was to describe a large cohort of patients with dialysis-requiring AKI derived from ordinary clinical practice within a large metropolitan area of an emerging country, assessing the impact of age and several clinical predictors on patient survival across the spectrum of human life.

**Methods:**

We analyzed registries drawn from 170 hospitals and medical facilities in Rio de Janeiro, Brazil, in an eleven-year period (2002–2012). The study cohort was comprised of 17,158 pediatric and adult patients. Data were analyzed through hierarchical logistic regression models and mixed-effects Cox regression for survival comparison across age strata.

**Results:**

Severe AKI was mainly hospital-acquired (72.6%), occurred predominantly in the intensive care unit (ICU) (84.9%), and was associated with multiple organ failure (median SOFA score, 11; IQR, 6–13). The median age was 75 years (IQR, 59–83; range, 0–106 years). Community-acquired pneumonia was the most frequent admission diagnosis (23.8%), and sepsis was the overwhelming precipitating cause (72.1%). Mortality was 71.6% and was higher at the age extremes. Poor outcomes were driven by age, mechanical ventilation, vasopressor support, liver dysfunction, type 1 cardiorenal syndrome, the number of failing organs, sepsis at admission, later sepsis, the Charlson score, and ICU admission. Community-acquired AKI, male gender, and pre-existing chronic kidney disease were associated with better outcomes.

**Conclusions:**

Our study adds robust information about the real-world epidemiology of dialysis-requiring AKI with considerable clinical detail. AKI is a heterogeneous syndrome with variable clinical presentations and outcomes, including differences in the age of presentation, comorbidities, frailty state, precipitation causes, and associated diseases. In the cohort studied, AKI characteristics bore more similarities to upper-income countries as opposed to the pattern traditionally associated with resource-limited economies.

## Introduction

Acute kidney injury (AKI) is a persistent health challenge associated with significant short- and long-term adverse consequences [1–3]. Once classified in relatively few categories, it is now clear that AKI is a syndrome that comes in many guises with varying risk factors, causes, clinical features, severity, and prognoses [4]. Although there is a fair amount of large-scale information on the epidemiology and features of AKI, the large administrative databases that enable the study of AKI at the population level are generally not collected for clinical research. This may limit the depth and breadth of the data content and, consequently, the ability to mirror real-world clinical practice [5, 6].

Significant knowledge gaps remain concerning the geographical variation of AKI and its differing outcomes [7]. Current information regarding AKI epidemiology in developing nations derives mainly from isolated centers with few quality, multicentric, epidemiological studies available. The International Society of Nephrology launched the 0by25 initiative almost a decade ago to eliminate or at least reduce avoidable AKI-related deaths worldwide by 2025 [1]. A stated purpose behind that endeavor was to generate more comprehensive information regarding the worldwide epidemiology of AKI.

This study reports on a large and detailed cohort of dialysis-requiring AKI sequentially drawn from ordinary clinical practice in hospitals and other health facilities within a large metropolitan area of an upper-middle-income economy. We aimed to gain insight into the clinical heterogeneity of urban AKI and assess the impact of age across the spectrum of human life, predisposing factors, comorbidities, frailty, precipitation causes, associated diseases, and concurrent organ failure on patient survival.

## Material and methods

The study protocol was approved by Pedro Ernesto University Hospital’s Institutional Review Board, which waived the need for informed consent based on the observational and non-interventional nature of the study. This manuscript adheres to the STROBE guidelines for cohort studies.

### Study design and settings

This was an 11-year (2002–2012) prospective, multicenter cohort study on the clinical characteristics and outcomes of AKI in the metropolitan area of Rio de Janeiro. It involved 170 hospitals and medical facilities of both private and public governance. Dialysis modalities included continuous automated peritoneal dialysis (PD) and hemodialysis with standard machinery in the following methods: conventional intermittent dialysis, prolonged intermittent renal replacement therapy (PIRRT), or PIRRT in continuous mode (C-PIRRT, meaning continuous hemodialysis provided with standard dialysis equipment and PIRRT set-up parameters) [8]. After nephrology referral and renal replacement therapy (RRT) indication, data was entered by attending nephrologists in the clinical module of NefroWeb, a customized Microsoft SQL server database (for further details of the NefroWeb database, medical institutions, and technical aspects of RRT, refer to, Sections 1.1 and 1.2 in S1 File).

### Participants, inclusion and exclusion criteria, and study size

**Fig 1:**
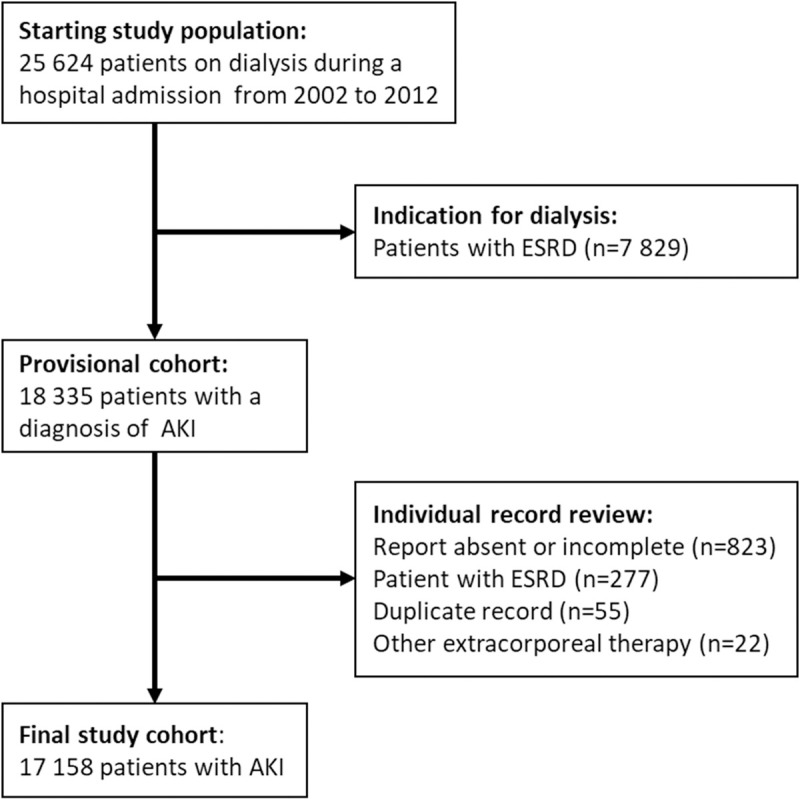
Study flowchart.

All adult or pediatric patients admitted to one of the 170 hospitals and medical facilities that required dialysis during hospitalization were eligible for inclusion in this cohort. Out of the 25,624 patients who were dialyzed, we excluded 7,289 patients diagnosed with end-stage kidney disease (either on previous chronic outpatient RRT or chronic kidney disease (CKD) patients commencing dialysis treatment at the time of hospital admission). After individual record review, we excluded an additional 1,177 patients with incomplete data. The final study cohort was comprised of 17,158 patients (Fig 1).

### Data collection and initial variables

We recorded the following demographic and clinical variables: age, sex, self-declared race, ICD-10 codes for the clinical diagnosis upon admission and the renal syndrome, comorbidities, precipitating causes of AKI, indications for RRT, and hospital governance (public or private). Detailed medical history and laboratory studies at enrollment identified underlying non-dialytic CKD. Therefore, patients were considered to have *de novo* acute kidney injury or acute-on-chronic kidney injury (ACKD) [9] (for further details of data collection and categorizations, refer to, Section 1.3 in S1 File). Variables regarding RRT prescription were also logged. Outcomes were in-hospital mortality and renal recovery status at discharge (dialysis dependence, partial or complete recovery). Recovery was defined as complete when serum creatinine returned to the same level as or a lower level than the enrollment value or partial when serum creatinine was lower than the peak but higher than the enrollment value [10].

### Database entries and review

After the first 3,506 patients, we performed an interim analysis to assess consistency and ascertain the need for adjustments in the data collection instrument. We noticed that many entries were amenable to subcategorization, enabling refinement in identifying the major diseases and risk factors associated with AKI development. We adjusted these entries retrospectively in the existing records, and from that date on, we expanded the database to prospectively record 80 non-mutually exclusive diagnostic groups, 17 comorbid conditions, 11 precipitation factors, six associated failing organs or systems, and six indications for the initiation of RRT (Section 1.3 in S1 File).

To further explore potential differences in age-associated clinical variables and outcomes, we stratified patient data into 13 age groups (0–2 months, two months to 1 year, 1–9 years, 10–19 years, and every subsequent decade until >100 years of age). Records were reviewed for implausible value, event ascertainment, adjudication, and data cleaning by a pediatric nephrologist (TLCY) and two adult nephrologists (CLRG, JHRS).

### Other inputs and definitions

We defined associated organ failure categorically by a simple organ failure count (SOFC) [11] and sequential organ failure assessment (SOFA) scoring (Section 1.3 in S1 File). The diagnosis of sepsis was based on the 2001 consensus definition [12], as ascertained by the attending physician. We defined community-acquired AKI as a dialysis requirement within the first 48 hours of hospital admission and confirmed it by record review. We considered later cases as hospital-acquired. Cardiorenal syndrome (CRS) type I was defined as an acute cardiac injury episode leading to AKI, and CRS type II was defined as an existing chronic abnormality in cardiac function that was followed by worsening kidney function [13]. The Charlson comorbidity score (CCS) and Elixhauser comorbidity score (ECS) were derived from the extensive list of comorbidities and clinical diagnoses available in the database and calculated using an R *comorbidity* package [14].

### Statistical analysis

Analyses were performed with the R statistical environment. Significance was set at p < 0.05. Categorical variables were summarized as the number and the percentage of observations and were compared with the chi-square test. Continuous variables were summarized as the mean and standard variation or median and interquartile range (IQR). They were compared with the Student *t*-test and the Mann-Whitney U test, as appropriate. We evaluated variables for their influence on the dichotomous outcome of survival or death during hospital admission. We adjusted p-values for multiple comparisons by the BH method (false discovery rate). We performed bivariate two-level hierarchical logistic regression to analyze factors associated with in-hospital mortality (the individual hospitals were the random effect variable), and we considered only variables with p < 0.10 for the multivariate regression analyses. After verification of collinearity (we excluded variables with an inflation variation factor > 5), variables selected for inclusion as fixed-effects in the two-level hierarchical multivariate logistic regression model were those with biological or plausible rationale according to current knowledge and included the following: demographic variables (age, sex, race), precipitating causes (sepsis at admission, later sepsis, liver dysfunction, CRS type I, hypovolemia, nephrotoxicity), settings (hospital governance, community vs. hospital-acquired AKI, medical admission, intensive care unit (ICU) admission), indication for dialysis (oliguria, hypervolemia, acidemia), comorbidities (pre-existing CKD, CCS, and ECS scores), and severity of presentation (mechanical ventilation, vasopressors, number of failing organs). We considered individual hospitals as the random effect variable. We selected the final model using a criterion-based approach by minimizing the Akaike information criterion and calculating the area under the receiver operating characteristic curve. Effect estimates are presented as odds ratio (OR) and 95% confidence interval (CI). To access the model’s calibration, we conducted the Hosmer-Lemeshow goodness of fit test on a non-hierarchical logistic regression model in which we included all the above covariates, including individual hospitals, as an additional fixed-effect. In addition to considering individual hospitals as random effects in the multilevel model and acknowledging the heterogenicity of medical institutions involved (i.e., the predominance of medium and small private medical facilities), we conducted an additional sensitivity analysis after stratifying patients according to hospital size (small, < 50 beds; medium, 51–150 beds; large, > 151 beds). Finally, considering the wide range of age distributions, from newborns to centenarians, we analyzed age-stratified hospital survival curves by the Kaplan-Meier method with the log-rank test. We also analyzed age-stratified survival by a mixed-effects Cox regression model adjusted for sex, race, AKI characteristics, and the CCS, considering individual hospitals as a random effect. We analyzed the Schoenfeld residuals to verify the model’s assumptions.

## Results

### Demographic characteristics and comorbidities (Table 1)

The median age was 75 years (IQR, 59–83; range, 0–106 years), 84.3% of the cohort was white, and 54.2% were males. The pediatric population (< 18 years) comprised only 3.8% of the cohort (Section 2, eTable 2a–2c in S1 File). Age distribution was stable across the study period (eFig 1a and 1b in S2 File). Arterial hypertension (43.6%), heart disease (32.4%), CKD (31.7%), diabetes mellitus (20.6%), and neoplastic disease (14.22%) were the most frequent comorbidities.

**Table 1:** Description of patient demography and other clinical aspects at hospital admission.

	All (n = 17158)	Survivors (n = 4870)	Non-survivors (n = 12288)	OR (95% CI)	p value ^a^
**Age**	75 (59–83)	67 (52–78)	77 (64–85)	1.02 (1.01–1.02)	p < 0.001
**Gender**					
Male	9302 (54.2%)	2862 (58.8%)	6440 (52.4%)	Ref.	
Female	7856 (45.8%)	2008 (41.2%)	5848 (47.6%)	1.29 (1.21–1.38)	p < 0.001
**Ethnicity**					
White	14218 (82.9%)	3864 (79.6%)	10314 (84.2%)	Ref.	
Afro-Brazilian	2877 (16.8%)	978 (20.1%)	1891 (15.4%)	0.82 (0.71–0.94)	p < 0.01
Asian	62 (0.4%)	14 (0.3%)	48 (0.4%)	ns	ns
Indigenous	1 (0.00%)	0 (0.00%)	1(0.00%)	ns	ns
**Hospital governance**					
Private	16020 (93.4%)	4418 (90.7%)	11602 (94.4%)	1.73 (1.52–1.95)	p < 0.001
Public	1138 (6.6%)	452 (9.3%)	686 (5.6%)	Ref.	
**Comorbidities**					
Arterial hypertension	7475 (43.6%)	2203 (45.2%)	5272 (42.9%)	0.90 (0.85–0.97)	p < 0.05
Heart disease	5555 (32.4%)	1561 (32.1%)	3994 (32.5%)	1.02 (0.95–1.09)	p = 0.739
Chronic kidney disease	5440 (31.7%)	1961 (40.3%)	3478 (28.3%)	0.58 (0.54–0.62)	p < 0.001
Diabetes mellitus	3539 (20.6%)	1115 (22.9%)	2424 (19.7%)	0.82 (0.76–0.89)	p < 0.001
Neoplastic disease	2437 (14.2%)	727 (14.9%)	1710 (13.9%)	0.92 (0.83–1.01)	p = 0.105
Peripheral arterial disease	1888 (11.0%)	519 (10.7%)	1369 (11.1%)	1.05 (0.94–1.17)	p = 0.425
Neurologic disease	1722 (10.0%)	334 (6.9%)	1388 (11.3%)	1.72 (1.52–1.96)	p < 0.001
Lung disease	1396 (8.1%)	237 (4.9%)	1159 (9.4%)	2.03 (1.76–2.35)	p < 0.001
Immobilization	724 (4.2%)	132 (2.7%)	592 (4.8%)	1.81 (1.50–2.20)	p < 0.001
Liver disease	671 (3.9%)	91 (1.9%)	580 (4.7%)	2.60 (2.09–3.27)	p < 0.001
Obesity	634 (3.7%)	204 (4.1%)	430 (3.5%)	0.82 (0.70–0.98)	p < 0.065
Immunodeficiency	513 (3.0%)	146 (3.0%)	367 (3.0%)	0.99 (0.82–1.21)	p = 0.969
**CCS (mean ± SD)**	2.02 ± 0.87	1.94 ± 0.88	2.05 ± 0.85	1.15 (1.11–1.20)	p < 0.001
**ESC (mean± SD**)	3.00 ± 1.46	2.89 ± 1.47	3.04 ± 1.42	1.07 (1.04–1.09)	p < 0.001

Data presented as median (IQR) or frequency; OR: odds ratio (univariate logistic regression); CCS: Charlson comorbidity score; ESC: Elixhauser comorbidity score; ICU: Intensive care unit; ns: non-significant.

^a^ p values adjusted for multiple comparisons by the FDR method.

### Hospital admission, associated diagnosis, and severity of presentation (Table 2)

Most of the AKI cases were admitted to medical services (75.1%) and initiated dialysis in an ICU (84.9%). Community-acquired pneumonia was the principal medical diagnosis (23.8%) followed by hospital-acquired pneumonia (15.9%), ischemic heart disease (9.0%), and heart failure (8.1%). Emergency abdominal surgery (4.4%), cardiac surgery (4.4%), and surgery of the spine-pelvis-hip-knee axis were the primary surgical diagnoses (eFig 2 in S2 File). Table 2 also shows the association of the main AKI-associated conditions with the odds of mortality. The cohort had a high disease severity: 74.8% of the patients were on mechanical ventilation, 70.7% were on vasopressor support, and the median SOFA score was 11 (IQR, 6–13). Isolated AKI occurred in 17.9% of the patients. Across all the 13 different age strata, AKI was predominantly associated with at least two concurrent failing organs, particularly in children aged under one year and in adults over 70 years of age (eFig 3 in S2 File).

**Table 2:** Hospital admission, associated diagnosis, and severity of presentation at the day of RRT initiation.

	All (n = 17158)	Survivors (n = 4870)	Non-survivors (n = 12288)	OR (95% CI)	p value ^a^
**Clinical setting (%)**					
Medical	12892 (75.1%)	3403 (69.9%)	9489 (77.2%)	Ref.	
Surgical	3186 (20.7%)	906 (20.7%)	2280 (20.7%)	1.00 (0.90–1.11)	p = 0.958
Obstetric	117 (0.7%)	79 (1.6%)	38 (0.3%)	0.19 (0.12–0.28)	p < 0.001
Urological	618 (3.5%)	402 (8.3%)	216 (1.8%)	0.21 (0.17–0.25)	p < 0.001
**ICU admission**	14710 (84.9%)	3729 (75.5%)	10981 (88.6%)	2.51 (2.30–2.73)	p < 0.001
**Main medical diagnoses** ^b^					
Community-acquired pneumonia	4081 (23.8%)	702 (14.4%)	3379 (27.5%)	2.25 (2.06–2.46)	p < 0.001
Hospital-acquired pneumonia	2722 (15.9%)	443 (9.1%)	2279 (18.6%)	2.27 (2.04–2.53)	p < 0.001
Ischemic heart disease	1550 (9.0%)	459 (9.4%)	1091 (8.9%)	0.93 (0.83–1.05)	p = 0.277
Acute decompensated heart failure	1387 (8.1%)	451 (9.3%)	936 (7.6%)	0.80 (0.71–0.90)	p < 0.001
Abdominal sepsis	1329 (7.7%)	304 (6.2%)	1025 (8.3%)	1.36 (1.19–1.56)	p < 0.001
Urinary tract infection	1302 (7.6%)	405 (8.3%)	897 (7.3%)	0.86 (0.76–0.98)	p < 0.06
Drug-induced nephrotoxicity	1208 (7.0%)	474 (9.7%)	734 (6.0%)	0.58 (0.52–0.66)	p < 0.001
Sepsis unknown/unspecified origin	1199 (7.0%)	231 (4.7%)	968 (7.9%)	1.71 (1.48–1.99)	p < 0.001
Cerebrovascular accident	1065 (6.2%)	216 (4.4%)	849 (6.9%)	1.59 (1.37–1.86)	p < 0.001
Skin and soft tissue sepsis	738 (4.3%)	222 (4.6%)	516 (4.2%)	0.91 (0.78–1.07)	p = 0.305
**Main surgical diagnoses** ^b^					
Abdominal emergency	758 (4.4%)	183 (3.8%)	575 (4.7%)	1.25 (1.06–1.49)	p < 0.05
Cardiac surgery	752 (4.4%)	257 (5.3%)	495 (4.0%)	0.75 (0.64–0.88)	p < 0.001
Spine-pelvis-hip-knee surgery	491 (2.9%)	105 (2.2%)	368 (3.1%)	1.47 (1.18–1.83)	p < 0.001
Elective surgery	269 (1.6%)	101 (2.1%)	168 (1.4%)	0.65 (0.51–0.84)	p < 0.001
Major trauma (except head)	254 (1.5%)	81 (1.7%)	173 (1.4%)	0.84 (0.64–1.10)	p = 0.261
Vascular surgery	246 (1.4%)	81 (1.6%)	165 (1.4%)	0.80 (0.61–1.05)	p = 0.127
Head trauma	208 (1.2%)	44 (0.9%)	164 (1.3%)	1.48 (1.07–2.09)	p < 0.06
Neurosurgery (non-trauma)	158 (0.9%)	32 (0.6%)	126 (1.0%)	1.56 (1.07–1.34)	p < 0.06
**Organ failure**					
Mechanical ventilation	12829 (74.8%)	2388 (49.0%)	10441 (85.0%)	5.87 (5.45–6.33)	p < 0.001
Vasopressor support	12122 (70.7%)	2178 (44.7%)	9944 (80.9%)	5.24 (4.87–5.60)	p < 0.001
Neurological	5913 (34.5%)	1043 (21.4%)	4870 (39.6%)	2.40 (2.23–2.60)	p < 0.001
Coagulation	1761 (10.3%)	311 (6.4%)	1450 (11.8%)	1.96 (1.72–2.23)	p < 0.001
Liver	1165 (6.8%)	195 (4,0%)	970 (7.9%)	2.05 (1.75–2.410	p < 0.001
Gastrointestinal	860 (5.0%)	175 (3.6%)	685 (5.6%)	1.58 (1.34–1.88)	p < 0.001
No additional failure	3066 (17.9%)	1993 (40.9%)	1073 (8.7%)	Ref.	
1 additional failure	1723 (10.0%)	699 (14.4%)	1024 (8.3%)	2.72 (2.41–3.07)	p < 0.001
2 additional failures	5787 (33.7%)	1154 (23.7%)	4633 (37.7%)	7.45 (6.76–8.23)	p < 0.001
≥ 3 additional failures	6582 (38.4%)	1024 (21.0%)	6582 (45.3%)	10.08 (9.12–11.14)	p < 0.001
**SOFA score** ^c^	11 (6–13)	7 (4–12)	12 (8–15)	1.14 (1.13–1.15)	p < 0.001

Data presented as median (IQR) or frequency; RRT: renal replacement therapy; OR: odds ratio (univariate logistic regression); CRS: cardiorenal syndrome; SOFA: sequential organ failure assessment.

^**a**^ p values adjusted for multiple comparisons by the FDR method.

^**b**^ Most frequent diagnoses out of 80 primary non-exclusively diagnostic categories.

^**c**^ The SOFA score was available for 9280 patients.

### AKI characteristics, precipitating causes, and RRT aspects (Table 3)

**Fig 2:**
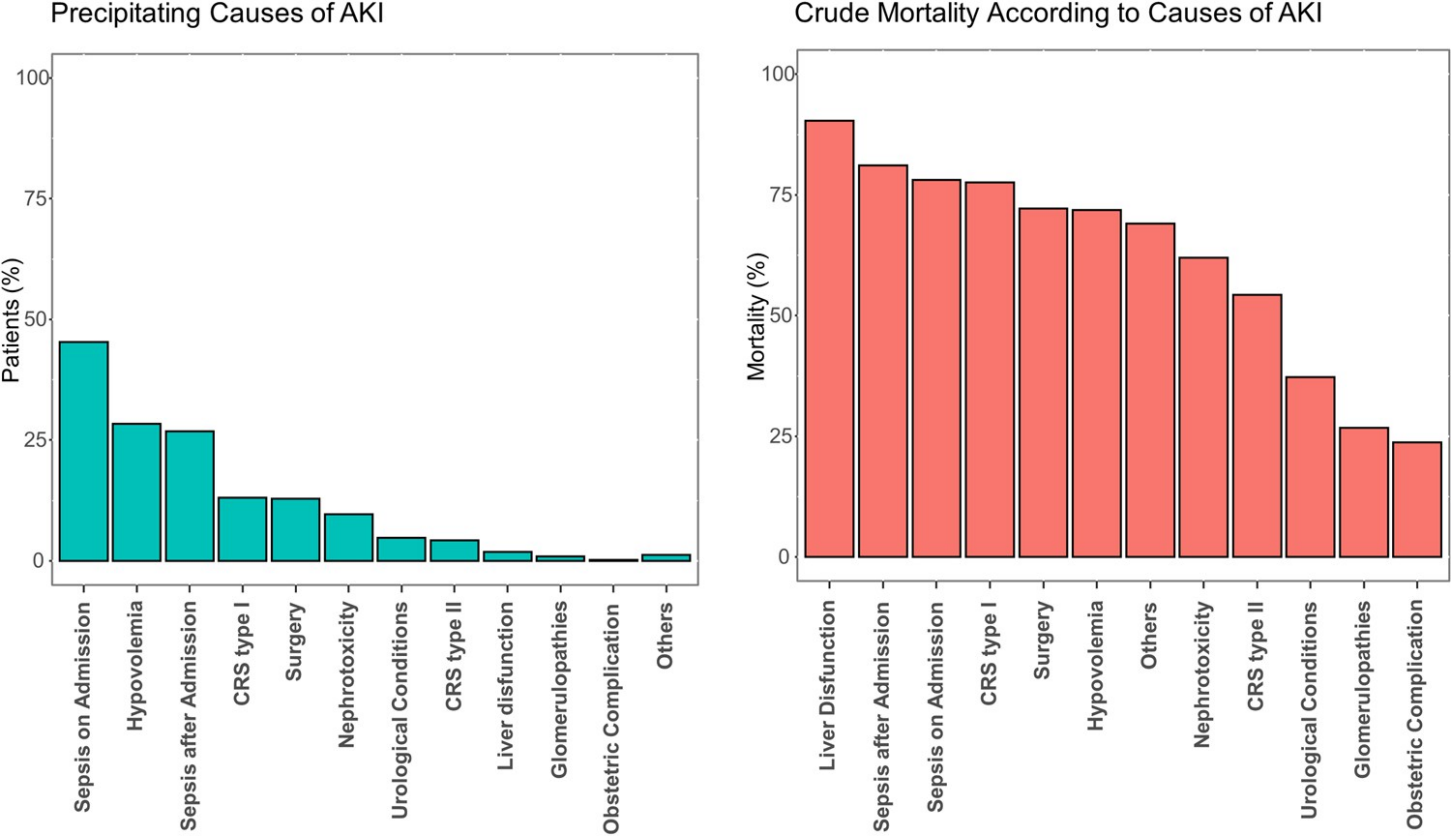
**a)** Precipitating causes of AKI and **b)** Crude mortality rates. Causes are not mutually exclusive. CRS: cardiorenal syndrome.

AKI was more frequently *de novo* (68.3%) and hospital-acquired (72.6%). Baseline CKD was more frequent in community-acquired AKI than in hospital-acquired AKI (42.3% vs. 27.7%, respectively). Sepsis was the most common precipitating cause both upon admission (45.3%) or further along the hospital course (26.8%) (Fig 2A). We observed significantly higher odds for mortality in patients with sepsis on admission and those that developed sepsis at a later stage (OR, 1.81; 95% CI, 1.69–1.94; and OR, 2.05; 95% CI, 1.84–2.18; respectively). Among the precipitating causes of AKI, liver dysfunction carried the highest risk for mortality (OR, 4.09; 95% CI, 2.84–6.13) (Fig 2B). Rates of sepsis and other precipitating causes of AKI were relatively stable across the 11-year period (eFig 4 in S2 File).

**Table 3:** AKI and RRT aspects at the day of dialysis initiation.

	All	Survivors	Non-survivors	OR	p value ^a^
	(n = 17158)	(n = 4870)	(n = 12288)	(95% CI)	
**AKI category**					
ACKD	5440 (31.7%)	1961 (40.3%)	3478 (28.3%)	Ref.	
*De novo* AKI	1178 (68.3%)	2909 (59.7%)	8809 (71.7%)	1.70 (1.59–1.82)	p < 0.001
**Setting**					
Hospital-acquired AKI	12460 (72.6%)	2721 (55.9%)	9739 (79.3%)	Ref.	
Community-acquired AKI	2698 (27.4%)	2149 (44.1%)	2549 (20.7%)	0.33 (0.30–0.35)	p < 0.001
**Precipitating causes of AKI**					
Sepsis on admission	7767 (45.3%)	1703 (35.0%)	6064 (49.4%)	1.81 (1.69–1.94)	p < 0.001
Hypovolemia	4880 (28.4%)	1373 (28.2%)	3507 (28.5%)	1.01 (0.94–1.09)	p = 0.650
Sepsis after admission	4598 (26.8%)	869 (17.8%)	3729 (30.4%)	2.05 (1.84–2.18)	p < 0.001
CRS type 1	2245 (13.1%)	504 (10.3%)	1741 (14.2%)	1.42 (1.29–1.58)	p < 0.001
Major surgery	2207 (12.9%)	614 (12.6%)	1593 (13.0%)	1.02 (0.93–1.14)	p = 0.546
Nephrotoxicity	1652 (9.6%)	628 (12.9%)	1024 (8.3%)	0.61 (0.55–0.68)	p < 0.001
Urinary tract obstruction	833 (4.9%)	522 (10.7%)	311 (2.5%)	0.21 (0.18–0.24)	p < 0.001
CRS type 2	727 (4.2%)	332 (6.8%)	395 (3.2%)	0.45 (0.39–0.52)	p < 0.001
Liver dysfunction	334 (2.0%)	32 (0.2%)	302 (1.8%)	4.09 (2.84–6.13)	p < 0.001
Glomerular disease	164 (1.0%)	120 (2.5%)	44 (0.4%)	0.14 (0.09–0.19)	p < 0.001
Obstetric complication	111 (0.7%)	75 (1.6%)	36 (0.3%)	0.12 (0.05–0.25)	p < 0.001
Other	194 (1.1%)	60 (1.2%)	134 (1.1%)	0.88 (0.65–1.20)	p = 0.456
1 factor	10381 (60.5%)	3164 (65.0%)	7217 (58.7%)	Ref.	
2 factors	5654 (33.0%)	1448 (29.7%)	4206 (34.2%)	1.27 (1.18–1.36)	p < 0.001
≥ 3 factors	1123 (6.6%)	258 (5.3%)	865 (7.0%)	1.46 (1.27–1.7)	p < 0.01
**Renal SOFA** ^b^					
Renal SOFA (mean ± SD)	3.35 ± 0.87	3.43 ± 0.83	3.28 ± 0.89	0.86 (0.82–0.91)	p < 0.01
Renal SOFA > 2 (points)	79.1%	82.5%	77.9%	0.74 (0.66–0.83)	p < 0.01
**Criteria for commencing RRT**					
Azotemia	14428 (84.1%)	4212 (86.5%)	10216 (83.1%)	0.77 (0.70–0.84)	p < 0.001
Oliguria	13007 (75.8%)	3142 (64.5%)	9865 (80.3%)	2.23 (2.08–2.41)	p < 0.001
Acidemia	12616 (73.5%)	3137 (64.4%)	9479 (77.1%)	1.86 (1.73–2.00)	p < 0.001
Fluid overload	6532 (38.1%)	1606 (33.0%)	4926 (40.1%)	1.36 (1.26–1.45)	p < 0.001
Hyperkaliemia	3552 (20.7%)	1111 (22.8%)	2441 (19.9%)	0.83 (0.77–0.90)	p < 0.001
Intoxication	15 (0.1%)	6 (0.1%)	9 (0.1%)	0.59 (0.21–1.77)	p = 0.356
Other	350 (2.0%)	123 (2.5%)	227 (1.9%)	0.72 (0.58–0.90)	p < 0.05
1 criterion	10381 (60.5%)	3164 (65%)	7217 (58,7%)	Ref.	
2 criteria	5654 (33%)	1448 (29.7%)	4206 (34.2%)	1.27 (1.18–1.36)	p < 0.001
≥ 3 criteria	1123 (6.5%)	258 (5.3%)	865 (7%)	1.47 (1.27–1.70)	p < 0.001
**Initial RRT modality** ^c^					
PIRRT	7993 (46.6%)	1567 (32.3%)	6426 (52.3%)	3.76 (3.49–4.07)	p < 0.001
IHD	5383 (31.4%)	2578 (52.9%)	2805 (22.4%)	Ref.	
C-PIRRT	3400 (19.8%)	586 (12.0%)	2814 (22.9%)	4.41 (3.98–4.89)	p < 0.001
PD	360 (2.1%)	130 (2.7%)	230 (1.9%)	1.62 (1.30–2.03)	p < 0.001
**Other RRT data**					
Hospital days before RRT	7 (2–16)	4 (2–10)	8 (3–18)	1.01 (1.01–1.02)	p < 0.001
RRT sessions	7 (3–15)	9 (3–14)	6 (3–14)	0.99 (0.99–0.994)	p < 0.001

Data presented as median (IQR) or frequency; AKI: acute kidney injury; RRT: renal replacement therapy; ACKD: acute on chronic kidney disease; CKD: chronic kidney disease; CRS: cardiorenal syndrome; SOFA: sequential organ failure assessment; PIRRT: prolonged intermittent renal replacement therapy; IHD: intermittent hemodialysis; C-PIRRT: PIRRT, continuous mode; PD: peritoneal dialysis.

^a^ p values adjusted for multiple comparisons by the FDR method.

^b^ The SOFA score was available for 9280 patients.

^c^ The Initial RRT modality information was available for 17136 patients.

The leading causes for initiating RRT were azotemia (84.1%), oliguria (75.8%), and acidosis (73.8%). The most prevalent initial treatment modality was PIRRT (45.3%). The median length of hospital stay before RRT initiation was seven days (IQR, 2–16).

### Overall mortality predictors

**Fig 3:**
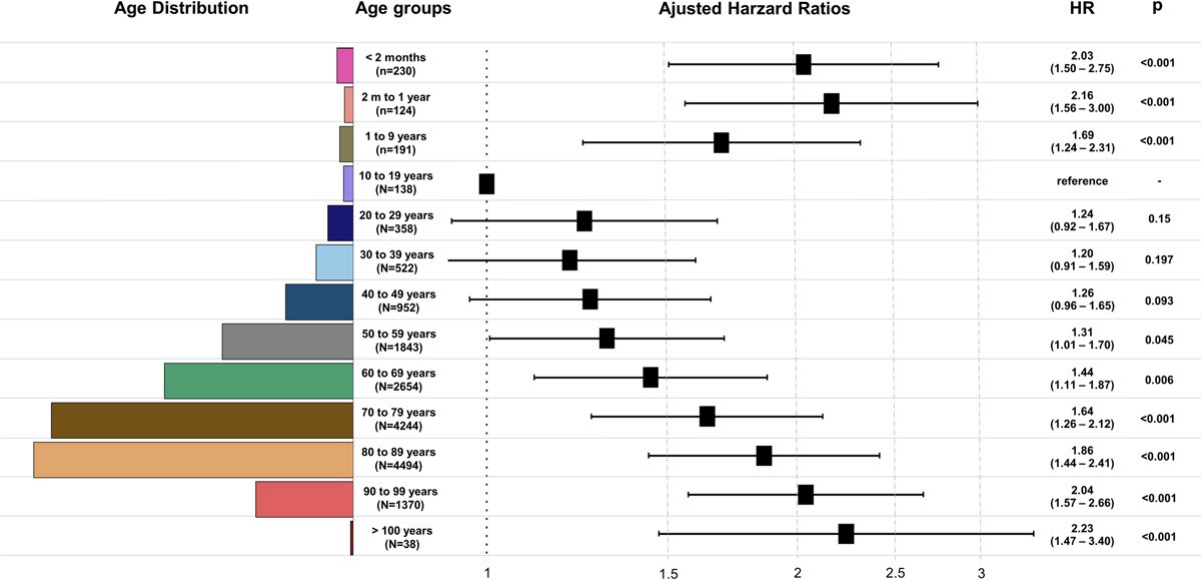
Age distribution in decades of life, number of patients according to 13 age strata, and hazard ratio for mortality based on a mixed-effects Cox proportional hazard model, stratified by decades of life and taking 10–19 years as reference. The model was adjusted for sex, AKI features (community x hospital-acquired, de novo x acute-on-chronic kidney disease, septic x non-septic AKI, oliguric x non-oliguric AKI), severity of presentation (number of failing organ), and comorbidities (Charlson comorbidity score). Individual hospitals variable was considered as a random-effect variable. Results are presented as a hazard ratio (HR) and 95% confidence intervals.

**Fig 4:**
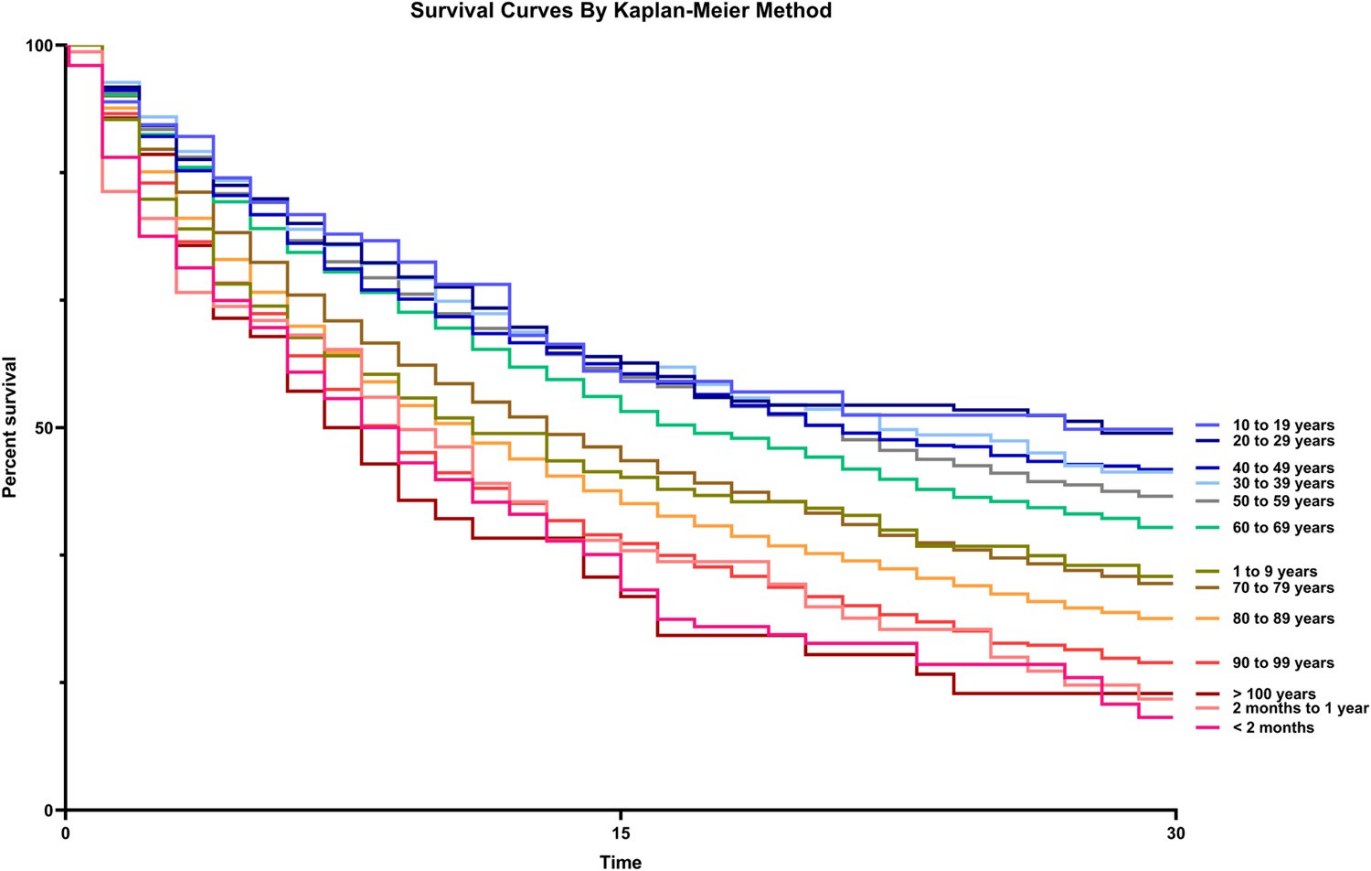
Survival curves according to Kaplan-Meier estimates, stratified by 13 age groups (log-rank test, p < 0.001).

Crude hospital mortality was 71.6% and remained relatively stable throughout the study period (eFig 5 in S2 File). The number of cases within each age stratum and their adjusted hazard ratio for mortality are depicted in Fig 3. Using the 10–19-year age group as a reference, mortality was significantly higher at both extremes of age, following a U-shaped pattern (Fig 3). Kaplan-Meier curves related to the same age strata are shown in Fig 4.

Several clinical variables that were significantly associated with mortality were identified by univariate analysis. The burden of underlying comorbidities, as summarized by the CCS (OR, 1.15; 95% CI, 1.11–1.20) and ECS (OR, 1.07; 95% CI, 1.04–1.09), was associated with worse survival. The median SOFA score was significantly higher in non-survivors (12; IQR, 8–15) than in survivors (7; IQR, 4–12). There was also an association between the number of associated failing organs and mortality. Patients with ≥ three failing organs had a much higher risk of mortality (OR, 10.08; 95% CI, 9.12–11.14), and 82.9% of non-survivors had at least two failing organs compared to 44.7% of survivors (eFigs 6 and 7 in S2 File). Patients with ACKD had lower mortality risks than those with *de novo* AKI (OR, 0.58; 95% CI, 0.54–0.62). Lower risk of mortality was also observed in patients with community-acquired AKI (OR, 0.33; 95% CI, 0.30–0.35).

Among the indications of RRT, oliguria was associated with the worst survival (OR, 2.23; 95% CI, 2.08–2.41) followed by acidosis (OR, 1.86; 95% CI, 1.73–2.00) and fluid overload (OR, 1.36; 1.26–1.45). Non-survivors had a longer length of hospital stay before RRT initiation (eight vs. four days; OR, 1.01; 95% CI, 1.01–1.02) and were more often initially treated with slow-extended modalities, such as PIRRT and C-PIRRT (73.8% vs. 38.9%, respectively; OR, 3.23; 95% CI, 2.22–4.64).

### Multivariate logistic model on mortality (Table 4)

Sixteen variables were retained in the multivariate analysis (Table 4). Those that were found to be associated with higher odds for mortality included: age, CCS, sepsis at admission, later sepsis, liver dysfunction, CRS type I, admission with a clinical diagnosis, ICU admission, the number of non-renal failing organs, vasopressor use, and mechanical ventilation. Oliguria was the only renal-related variable that was associated with a worse outcome. Male gender, community-acquired AKI, and ACKD were associated with better odds for survival. Hospital governance did not impact the outcome. A sensitive analysis stratified by hospital size showed that this variable did not affect the model assumptions (Section 3, eTable 3a-3c in S1 File).

**Table 4:** Two-level multivariate logistic regression on mortality.

	OR	(95% CI)	p value
Age (per year)	1.02	(1.01 to 1.02)	p < 0.001
Male gender	0.87	(0.80 to 0.94)	p < 0.001
Sepsis on admission	1.55	(1.39 to 1.73)	p < 0.01
Later sepsis	1.44	(1.27 to 1.63)	p < 0.001
Liver dysfunction	4.37	(2.86 to 6.68)	p < 0.001
CRS type I	1.22	(1.06 to 1.41)	p < 0.01
Public hospital	1.14	(0.87 to 1.49)	p = 0.336
Community-acquired AKI	0.61	(0.55 to 0.68)	p < 0.001
ICU admission	1.48	(1.31 to 1.67)	p < 0.001
Clinical admission	1.36	(1.23 to 1.67)	p < 0.001
Oliguria	1.48	(1.35 to 1.63)	p < 0.001
ACKD	0.88	(0.81 to 0.97)	p < 0.05
Charlson score (per point)	1.08	(1.01 to 1.16)	p < 0.05
Mechanical ventilation	1.52	(1.32 to 1.76)	p < 0.001
Vasopressors	1.46	(1.27 to 1.69)	p < 0.001
Non-renal organ failure (per organ)	1.40	(1.31 to 1.50)	p < 0.001

CRS: cardiorenal syndrome; ACKD: acute-on-chronic kidney disease; ICU: intensive care unit. Chi2 goodness-of-fit model test: p = 0.174; Model accuracy: 0.788 (95% CI: 0.776 to 0.791); Model sensitivity: 0.923; Area under the receiver-operating characteristic curve (c statistic): 0.797 (95% CI: 0.790 to 0.805). Hosmer–Lemeshow goodness-of-fit test: p = 0.233 (non-hierarchical logistic regression model with individual hospital as a fixed effect).

### Outcome in survivors

Among the 4,870 surviving patients, 26.7% completely recovered, 33.0% were discharged with partial recovery, and 23.3% remained dialysis-dependent. Unknown information on kidney function at discharge was observed in 17.0% of the patients. Those with ACKD were more likely to be discharged on dialysis (13.5% vs. 9.8%, p < 0.001) (eFig 8 in S2 File). Patients discharged on dialysis also presented with lesser multiorgan involvement than those that became dialysis-independent. Only 12.2% of those that ultimately left the hospital on RRT had ≥ three failing organs at admission as opposed to 20.8 and 23.9% of those with partial and complete recovery, respectively (p < 0.001). Most patients discharged with dialysis dependency were submitted to conventional intermittent dialysis (28.7%) compared to PIRRT, C-PIRRT, and continuous automatized PD (17.8%, 14.7%, and 22.3%, respectively) (Table 4 in S1 File).

## Discussion

This cohort study of 17,158 patients admitted to 170 medical facilities is one of the most extensive prospective investigations regarding the causes, clinical characteristics, and outcome of severe, dialysis-requiring AKI. Furthermore, it addresses the perceived need to increase the representation of developing countries in the global epidemiological knowledge base on AKI [1, 7]. Consistent with previous research [15–19], we found that dialysis-requiring AKI is associated with significant clinical adversity and a high mortality risk. Mounting evidence indicates that AKI in itself is a distinct, directly attributable risk factor for patient death [20–22]. However, the mortality risk is also significantly compounded by the severity of underlying acute medical conditions and pre-existing comorbidities. These factors are multiple, heterogeneous, temporarily variable, and difficult to account for [21]. A significant strength of this investigation is the size and detail of the database, which allowed this heterogeneity to be addressed by extensive detailing of associated diagnoses, comorbidities, precipitating factors, and severity of illness, as well as the power to ascertain age-related differences in a fashion not previously considered in other studies.

Out of the 80 categorized diagnoses in the database, three-fourths were medical conditions with a striking predominance of pulmonary infections both at admission or acquired during the hospital stay. These were involved in virtually 40% of AKI cases and were associated with increased odds for mortality. Major adverse cardiovascular events (ischemic heart disease, heart failure, and cerebrovascular accidents) were also frequently associated with severe AKI development. Postoperative AKI, whose incidence did not significantly differ between survivors and non-survivors, was responsible for one-fifth of the cases. However, the odds of death were higher for AKI that developed in the setting of abdominal emergency surgery, orthopedic surgery of the spine–pelvis–hip–knee axis, head trauma, and non-trauma neurosurgery. AKI development in urological and obstetric settings was responsible for ≤ 5% of the cases and portended a more favorable prognosis.

There was a high prevalence of nonrenal-associated organ dysfunction, with more than 70% of the patients requiring mechanical ventilation and vasopressor support. This clinical profile was analogous to the AKI features observed in other urban series [1, 9, 16, 17, 19]. Hospital-acquired AKI comprised approximately two-thirds of the cohort, once again matching the pattern noted in other large-scale prospective studies [9, 17].

We sought to refine and extend previous observations that analyzed children and adults as separate cohorts [7, 15–17, 23] by stratifying the survival analysis across 13 age groups encompassing the entire length of human life, from premature newborns to centenarians, revealing a clear U-shaped pattern of AKI-related mortality, with the highest rates at the extremes of age. This conformation persisted after multivariable adjustment.

Despite the extended age spectrum, the study participants’ median age (75 years) was 10–15 years higher than that reported in other similar studies [15–17, 24]. This feature might indicate a more vulnerable cohort, and age was an independent risk factor for the mortality of adults, increasing progressively at each age stratum [15].

Also, in agreement with preceding large series [17, 18, 25], sepsis was the most prevalent precipitating factor for AKI and was associated with a high risk of mortality either when diagnosed at admission or when acquired later. In a secondary analysis of the BEST Kidney Study, in-hospital mortality was significantly higher for septic AKI than for non-septic AKI (70.2% vs. 51.8%, respectively) [26]. We also observed this pattern in our study.

The combination of sepsis, advanced age, delayed development of AKI during hospital stay, oliguria, and severity of clinical presentation (herein reflected by higher SOFA scores and associated organ failure) indicated a particular group of patients with very poor prognosis, exposing the high mortality rate that this and other studies have documented [15–19].

The reported prevalence of RRT dependence after an episode of severe AKI is highly variable. The prevalence was 13.8% in the multicenter BEST kidney study [17] and 8.2% in the recently completed worldwide Standard versus Accelerated Initiation of Renal-Replacement Therapy in Acute Kidney Injury (STARRT-AKI) trial [24]. When developing and developed countries were compared, dialysis dependency at discharge after an AKI episode was 18.5% versus 5.7%, respectively [19]. In this study, there was a high prevalence of patients discharged on chronic dialysis (23.3%) and a significant association of pre-existing CKD with this outcome. This may be explained in part by the higher median age of the cohort. However, ACKD was associated with better survival odds, and patients ultimately discharged on chronic dialysis had less severe disease at presentation than those with partial or complete recovery of renal function. It has been previously shown that a smaller number and severity of acute insults are required for the development of AKI in patients with pre-existing CKD [27], suggesting that this subgroup might have suffered a lesser organ injury burden.

Our findings must be interpreted in the context of the study’s limitations. First, in comparison to most series, the case mortality rate was certainly overrated, as the focus was on severe dialysis-requiring AKI rather than on earlier stages. Second, despite the various design and analytic approaches, residual and unmeasured confounding variables cannot be ruled out, as in any observational study. Despite highly significant statistical associations between covariates and the outcome, a causal relationship cannot be assumed.

A third limitation was the lack of uniform policy for the indication and timing of dialysis, as this was an observational cohort investigation based on real-world clinical practice. These decisions were at the discretion of the nephrologists that contributed to the study. It is assumed that they followed standard criteria for commencing RRT, as indicated by more than one-fourth of the patients being dialyzed without delay within a 48-hour timeframe after hospital admission and conventional indications for starting dialysis recorded in 98% of the entries.

The balance between the optimal and the feasible is one of the main challenges in prospective cohort studies. To encourage enrollment, the NefroWeb clinical module was devised to minimize physician overburdening, as they were already busily involved with patient care. Therefore, the emphasis was on recording categorical data elements collected in daily practice through the extensive use of checkboxes and drop-down menus as opposed to numerical clinical variables, such as vital signs and laboratory measures. For example, this was applied in the definition of organ failure, which was extracted from the SOFA score and SOFC instrument at the bedside. The lack of precise numerical data may have resulted in loss of information and lower predictive ability and may be a fourth limitation of this study. However, the large size of the cohort, density of the collected categorical data, and innovative age-stratified analysis allowed for a depth of detail regarding the predisposing, comorbid, and risk factors for dialysis-requiring AKI that is the key milestone of the study.

Although the analysis did not identify hospital size as significantly influencing survival, the institutions differed in staffing, availability of resources and technology, and probably in overall quality of care, all of which ultimately might have an unmeasured impact on survival. These factors may present as a fifth limitation of this study. The painstaking process of individual record review, including individual hospitals as random effects in the multivariate model, and sensitivity analysis that considered hospital size were employed to minimize some of these inherent weaknesses.

It was not our intention to produce a population-level analysis regarding the incidence and prevalence of dialysis-requiring AKI. The institutions involved do not represent the whole scope of Rio de Janeiro’s medical facilities, as can be gathered by the higher prevalence of private hospitals in the cohort. Likewise, the results do not entirely reflect the complex diversity of disease burden in the whole of Brazil, considering that the metropolitan area of Rio de Janeiro is generally more urbanized and has a better healthcare infrastructure compared to some other regions in the country [28]. Finally, the absence of long-term follow-up of survivors represents an additional weakness of our cohort.

Conversely, the strength of this study is the large database prospectively derived from the real-world clinical practice of patients with dialysis-requiring AKI instead of centers of excellence or trials with strict selection criteria. This type of design allows for data collection with considerable clinical detail and may provide valuable information regarding the range and distribution of patients with a particular disease condition [29]. More broadly generalizable and applicable results to clinical care may also be produced, uncovering new opportunities for prevention while also assisting in selecting potential targets for improved resource allocation. Finally, we addressed the perceived knowledge gap about the worldwide epidemiology of AKI from the point of view of a middle-income country [7]. It is fitting to point out that despite drawing the cohort from a metropolitan area of an emerging economy, the AKI characteristics were akin to that of high-income countries, in contradiction to the pattern often associated with middle-income economies.

In conclusion, older individuals with significant comorbidities predominated in this cohort, although both extremes of age drove similarly poor outcomes. On multivariate analysis, mechanical ventilation, vasopressor support, liver dysfunction, the number of failing organs, sepsis at admission, later sepsis acquisition, the Charlson score, male gender, and age were independently associated with mortality. Oliguria was the only renal-related variable associated with poor prognosis. Favorable outcomes were associated with the male gender, ACKD, and community-acquired AKI.

## Conclusions

Our results endorse that dialysis-requiring AKI is a significant and devastating syndrome. It also highlights its significant clinical heterogeneity, including differences in the age of presentation, predisposing factors, comorbidities, frailty state, etiology, precipitation causes, associated diseases, concurrent organ failure, and response to therapy. Most past investigations have summarized patient data into relatively few groups or classes to analyze small datasets. As we have shown, there is a richness in the details that is lost with this exercise. We suggest that research efforts should acknowledge the complexity of AKI and use today’s ever-increasing large databases to provide more detailed profiling of individual patients. The combination of more extensive data collection with current advances in technology and data science may ultimately result in better individualized risk assessment and refinements in the process of care.

## Supporting information

S1 File Supplementary methods and tables. (DOCX)

S2 File Supplementary figures. (DOCX)

S1 Data Anonymized dataset of the study. (CSV)
