# Supplementary material for: A real-world prospective study on dialysis-requiring acute kidney injury
Source: PLoS One. 2022 May 5;17(5):e0267712. doi: 10.1371/journal.pone.0267712 (PMC9071163; doi:10.1371/journal.pone.0267712)
Supplement: S1 File — (DOCX) [file pone.0267712.s001.docx]

**S1 -** Supplementary Methods and Tables

**1. The NefroWeb**^®^ **database and dialytic methods**

The web-based NefroWeb® is a relational database developed for administrative purposes using the Microsoft SQL Server management system. It was designed to support a privately outsourced mobile inpatient dialysis enterprise in a wide range of public and private hospitals in the metropolitan area of Rio de Janeiro, Brazil. A clinical and epidemiological module was later added to the original database.

Each attending nephrologist was assigned a unique user ID and password to gain access to the database. The individual record of each incident patient contained all the variables described in the Methods section, along with a structured written report. RRT initiation date corresponded to the time zero, which was used as the primary reference for the study. The records were anonymized, exported to Microsoft Excel, and individually reviewed for consistency and categorization of variables before exporting for statistical analysis.

After the preliminary analysis and database expansion described in the Methods section, every new record included checklists containing at least one primary diagnostic group, one renal diagnosis, an extensive choice of comorbidities, and clinical setting details. This uniformity of date collections implies that, despite the potential differences in hospital complexity and specialisation, staffing, number of beds, access to state-of-the-art technology, and overall quality of care, the final database was remarkably consistent.

Dialysis modalities included continuous automated peritoneal dialysis and haemodialysis with standard machinery in following methods: conventional intermittent dialysis, prolonged intermittent renal replacement therapy (PIRRT), or PIRRT in continuous mode (C-PIRRT, meaning continuous haemodialysis provided with standard dialysis equipment and PIRRT set-up parameters). In our part of the world, continuous renal replacement therapy (CRRT) is 3 to 6 times more expensive than PIRRT or C-PIRRT, and many healthcare providers do not pay for it. Also, during the study period, our outsourcing dialysis company that attended the institutions that comprised the cohort had not yet sought approval from the country’s regulatory authorities to provide CRRT. Therefore, volume overloaded or hemodynamically unstable patients that required RRT were treated with PIRRT or C-PIRRT. Of note, only a few other medical institutions in Rio de Janeiro performed CRRT with equipment provided by a different dialysis company during that period.

Of note, no compelling evidence for the superiority of one mode of RRT above the other has been provided, and KDIGO guidelines suggest that the choice of RRT modality must be tailored to the available resources of the attending institution.

**1.2 Involved medical institutions**

During the 11-years period, our medical group provided high-quality nephrological support for a total of 170 medical institutions in the metropolitan region of Rio de Janeiro. There was a predominance of private hospitals and medical facilities that corresponded to 82% of total institutions (n=139 private institutions), and the representativity of public institutions (18%, n=31 public institutions) was lower than the actual distribution in Rio de Janeiro. eTable 1 depicts the characteristics of involved institutions. However, this does not invalidate our findings because it was not our intention to conduct a population-based analysis or track longitudinal changes in incidence and prevalence. Our primary objective was to describe overall characteristics of dialysis-requiring AKI in a large cohort of patients, and we addressed this in the discussion section of the manuscript.

Table 1. Characteristics of involved institutions

|  | All (n=170) | Private (n=139; 81.8%) | Public (n=31; 18.2%) |
| --- | --- | --- | --- |
| **Size (number of beds)** |  |  |  |
| Small (< 50) | 57 (33.5%) | 57 (41%) | 0 (0%) |
| Medium (51-150) | 72 (42.4%) | 62 (44.6%) | 10 (32.3%) |
| Large (> 151) | 41 (24.1%) | 20 (14.4%) | 21 (67.7%) |
| **Especiality** |  |  |  |
| General | 119 (70.0%) | 106 (76.3%) | 13 (41.9%) |
| Pediatric | 14 (8.2%) | 11 (7.9%) | 3 (9.7%) |
| Cardiology | 13 (7.6%) | 11 (7.9%) | 2 (6.5%) |
| Emergency/Trauma | 11 (6.5%) | 3 (2.2%) | 8 (25.8%) |
| Obstetric | 8 (4.7%) | 7 (5.0%) | 1 (3.2%) |
| Oncology | 3 (1.8%) | 1 (0.7%) | 2 (6.5%) |
| Infectious Diseases | 2 (1.2%) | 0 (0%) | 2 (6.5%) |

**1.3 Data acquisition, categorisations, indication of dialysis and organ failure definition**

The attending nephrologist was responsible for patient evaluation, the indication of dialysis and the appropriate method, and collecting the relevant data for feeding the database. We conducted these procedures on the day of dialysis indication by reviewing medical reports, physical examination, and laboratory results. The collection of a considerable amount of data, including raw data, would substantially increase the medical practice's daily burden. Therefore, we dichotomised most of our data into categories, including comorbidities and the presence of pre-existing CKD (a data imputed by attending nephrologist according to medical history, examination of pre-existing laboratory results, if available, and caregiver consultation). The recording of organ failures, for instance, was also a process based on clinical judgment, on-site laboratory review, and current medical practice and knowledge, resulting in categorisation of organ failure and SOFA scoring computing. According to classical indications (e.g., refractory hyperkalaemia, hypervolemia, severe acidaemia, azotaemia) or clinical judgment (e.g., progressive fluid overload), the initiation of dialysis was at the discretion of the attending nephrologist. The coding of precipitating causes of AKI was also based on clinical judgment at the bedside, medical history and chart review, physical examination, laboratory tests, and image studies mirroring the real-life aspects of nephrology actuation. Based on clinical judgment and laboratory review, this sort of medical evaluation was also applied for other categorisations, such as the definitions of precipitating cause of AKI (e.g., sepsis, hypovolaemia, CRS type I). As stated in the Methods section, we recorded 80 non-mutually exclusive diagnostic categories involved, directly or indirectly, in the AKI pathogenesis. We described only the ten most frequent clinical situations and the five most frequent surgical situations in Table 2.

**2 Supplemental Tables – Paediatric group**

Participants ≤ 18 years of age, the paediatric group, comprised 666 (3.8%) of the entire cohort from premature new-borns to late adolescents. The crude mortality rate of this segment was 63.3%. The overall presentation was very severe, with ≥ 90% of patients admitted to the ICU and ≥ 70% with more than two failing organs. The following tables describe the general aspects of this group.

Table 2a. Patient demographic and clinical aspects at hospital admission

| Variable | Levels | All (n=666) | Survivors (n=244) | Non-Survivors (n=422) | p |
| --- | --- | --- | --- | --- | --- |
| **Age** | Mean (SD) | 3.7 (5.5) | 5.0 (6.2) | 2.9 (5.0) | <0.001 |
| **Gender** | Female | 227 (34.1) | 81 (33.2) | 146 (34.6) | 0.778 |
|  | Male | 439 (65.9) | 163 (66.8) | 276 (65.4) |  |
| **Ethnicity** | Asian | 3 (0.5) | 0 (0.0) | 3 (0.7) | 0.397 |
|  | White | 482 (72.6) | 179 (73.7) | 303 (72.0) |  |
|  | Afro-Brazilian | 179 (27.0) | 64 (26.3) | 115 (27.3) |  |
| **Hospital Governance** | Private | 508 (76.3) | 182 (74.6) | 326 (77.3) | 0.494 |
|  | Public | 158 (23.7) | 62 (25.4) | 96 (22.7) |  |
| **Comorbidities** |  |  |  |  |  |
| Heart Disease |  | 142 (21.3) | 46 (18.9) | 96 (22.7) | 0.278 |
| Prematurity |  | 90 (13.5) | 25 (10.2) | 65 (15.4) | 0.079 |
| Chronic neuropathy |  | 66 (9.9) | 27 (11.1) | 39 (9.2) | 0.532 |
| Neoplasia |  | 63 (9.5) | 24 (9.8) | 39 (9.2) | 0.908 |
| Immunodeficiency |  | 27 (4.1) | 9 (3.7) | 18 (4.3) | 0.873 |
| Lung Disease |  | 21 (3.2) | 7 (2.9) | 14 (3.3) | 0.929 |
| Hypertension |  | 12 (1.8) | 6 (2.5) | 6 (1.4) | 0.505 |
| Immobility |  | 9 (1.4) | 5 (2.0) | 4 (0.9) | 0.402 |
| Liver disease |  | 3 (0.5) | 0 (0.0) | 3 (0.7) | 0.472 |
| Obesity |  | 4 (0.6) | 2 (0.8) | 2 (0.5) | 0.971 |
| CCS | 1 | 463 (69.5) | 164 (67.2) | 299 (70.9) | 0.366 |
|  | 2 | 182 (27.3) | 72 (29.5) | 110 (26.1) |  |
|  | 3 | 18 (2.7) | 8 (3.3) | 10 (2.4) |  |
|  | 4 | 3 (0.5) | 0 (0.0) | 3 (0.7) |  |
| EES | Mean (SD) | 1.9 (1.0) | 1.8 (0.9) | 2.0 (1.1) | 0.011 |

CCS: Charlson comorbidity score: EES: Elixhause score

Table 2b. Clinical aspects at the day of RRT initiation

| Variable | All (n=666) | Survivors (n=244) | Non-Survivors (n=422) | p |
| --- | --- | --- | --- | --- |
| **Clinical Setting (%)** |  |  |  |  |
| Medical | 482 (72.4) | 179 (73.4) | 303 (71.8) | 0.731 |
| Surgical | 150 (22.5) | 50 (20.5) | 100 (23.7) | 0.391 |
| Urological | 9 (1.4) | 5 (2.0) | 4 (0.9) | 0.402 |
| Trauma | 21 (3.2) | 8 (3.3) | 13 (3.1) | 1.000 |
| **ICU admission (%)** | 628 (94.3) | 229 (93.9) | 399 (94.5) | 0.841 |
| **Main medical or surgical diagnosis (%)** |  |  |  |  |
| Community acquired pnemonia | 133 (20.0) | 43 (17.6) | 90 (21.3) | 0.293 |
| Congenital Heart Disease | 131 (19.7) | 44 (18.0) | 87 (20.6) | 0.480 |
| Heart surgery | 100 (15.0) | 33 (13.5) | 67 (15.9) | 0.480 |
| Neonatal sepsis | 68 (10.2) | 17 (7.0) | 51 (12.1) | 0.049 |
| Hospital acquired pnemonia | 49 (7.4) | 14 (5.7) | 35 (8.3) | 0.288 |
| Unspecified sepsis | 40 (6.0) | 14 (5.7) | 26 (6.2) | 0.958 |
| Hematological neoplasia | 38 (5.7) | 12 (4.9) | 26 (6.2) | 0.622 |
| Neonatal Hypoxia | 34 (5.1) | 10 (4.1) | 24 (5.7) | 0.475 |
| Abdominal sepsis | 28 (4.2) | 10 (4.1) | 18 (4.3) | 1.000 |
| Drug induced nephrotoxicity | 28 (4.2) | 15 (6.1) | 13 (3.1) | 0.089 |
| Meningitis encephalitis | 27 (4.1) | 10 (4.1) | 17 (4.0) | 1.000 |
| Glomerulopathies | 25 (3.8) | 20 (8.2) | 5 (1.2) | <0.001 |
| Sepsis immunocompromised | 24 (3.6) | 8 (3.3) | 16 (3.8) | 0.899 |
| Thrombotic microangiopathy | 24 (3.6) | 14 (5.7) | 10 (2.4) | 0.042 |
| Diarrhea | 23 (3.5) | 15 (6.1) | 8 (1.9) | 0.007 |
| Neurosurgery | 3 (0.5) | 1 (0.4) | 2 (0.5) | 1.000 |
| **Organ Failure** |  |  |  |  |
| Mechanical Ventilation | 559 (83.9) | 168 (68.9) | 391 (92.7) | <0.001 |
| Vasopressors | 512 (76.9) | 142 (58.2) | 370 (87.7) | <0.001 |
| Neurological | 211 (31.7) | 59 (24.2) | 152 (36.0) | 0.002 |
| Coagulation | 157 (23.6) | 32 (13.1) | 125 (29.6) | <0.001 |
| Liver | 89 (13.4) | 19 (7.8) | 70 (16.6) | 0.002 |
| Grastointestival | 54 (8.1) | 18 (7.4) | 36 (8.5) | 0.705 |
| Failures |  |  |  |  |
| 0 | 73 (11.0) | 54 (22.1) | 19 (4.5) | <0.001 |
| 1 | 62 (9.3) | 43 (17.6) | 19 (4.5) |  |
| 2 | 221 (33.2) | 73 (29.9) | 148 (35.1) |  |
| >3 | 310 (46.5) | 74 (30.3) | 236 (55.9) |  |
| **SOFA (Mean (SD))** | 9.9 (5.6) | 7.6 (4.8) | 12.8 (5.4) | 0.003 |

SOFA: Sequential organ failure assessment

Table 2c. AKI and RRT aspects at the day of dialysis initiation

| Variable | All (n=666) | Survivors (n=244) | Non-Survivors (n=422) | p |
| --- | --- | --- | --- | --- |
| **AKI Phenotype1** |  |  |  |  |
| De novo AKI | 591 (88.7) | 208 (85.2) | 383 (90.8) | 0.041 |
| ACKD | 75 (11.3) | 36 (14.8) | 39 (9.2) |  |
| **AKI Phenotype2** |  |  |  |  |
| Hospital acquired | 471 (70.7) | 140 (57.4) | 331 (78.4) | <0.001 |
| Community acquired | 195 (29.3) | 104 (42.6) | 91 (21.6) |  |
| **Precipitating causes of AKI** |  |  |  |  |
| Sepsis on admission | 321 (48.2) | 104 (42.6) | 217 (51.4) | 0.035 |
| Hypovolemia | 162 (24.3) | 53 (21.7) | 109 (25.8) | 0.273 |
| Later sepsis | 148 (22.2) | 38 (15.6) | 110 (26.1) | 0.002 |
| CRS type I | 142 (21.3) | 38 (15.6) | 104 (24.6) | 0.008 |
| Surgery | 126 (18.9) | 45 (18.4) | 81 (19.2) | 0.892 |
| Nephrotoxicity | 49 (7.4) | 20 (8.2) | 29 (6.9) | 0.633 |
| Urological Condictions | 15 (2.3) | 9 (3.7) | 6 (1.4) | 0.103 |
| CRS type II | 4 (0.6) | 2 (0.8) | 2 (0.5) | 0.971 |
| HRS | 4 (0.6) | 0 (0.0) | 4 (0.9) | 0.315 |
| Glomerular diseases | 24 (3.6) | 21 (8.6) | 3 (0.7) | <0.001 |
| Number of causes |  |  |  |  |
| 1 | 390 (58.6) | 165 (67.6) | 225 (53.3) | 0.003 |
| 2 | 242 (36.3) | 71 (29.1) | 171 (40.5) |  |
| 3 | 31 (4.7) | 7 (2.9) | 24 (5.7) |  |
| 4 | 3 (0.5) | 1 (0.4) | 2 (0.5) |  |
| **Criteria for commencing RRT** |  |  |  |  |
| Oliguria | 590 (88.6) | 196 (80.3) | 394 (93.4) | <0.001 |
| Acidosis | 403 (60.5) | 123 (50.4) | 280 (66.4) | <0.001 |
| Hypervolemia | 387 (58.1) | 131 (53.7) | 256 (60.7) | 0.094 |
| Hyperkalemia | 173 (26.0) | 56 (23.0) | 117 (27.7) | 0.207 |
| Azotemia | 421 (63.2) | 165 (67.6) | 256 (60.7) | 0.087 |
| Number of indications |  |  |  |  |
| 1 | 61 (9.2) | 32 (13.1) | 29 (6.9) | 0.008 |
| 2 | 165 (24.8) | 66 (27.0) | 99 (23.5) |  |
| 3 | 231 (34.7) | 88 (36.1) | 143 (33.9) |  |
| 4 | 131 (19.7) | 35 (14.3) | 96 (22.7) |  |
| 5 | 76 (11.4) | 23 (9.4) | 53 (12.6) |  |
| 6 | 2 (0.3) | 0 (0.0) | 2 (0.5) |  |
| **Initial RRT modality^a^** |  |  |  |  |
| PD | 326 (48.9) | 118 (48.4) | 208 (49.3) | 0.001 |
| CVVHD | 215 (32.3) | 58 (23.8) | 157 (37.2) |  |
| HDI | 54 (8.1) | 37 (15.2) | 17 (4) |  |
| SLED | 64 (9.6) | 29 (11.9) | 35 (8.3) |  |
| **Other RRT data** |  |  |  |  |
| Days_pre_RRT Mean (SD) | 10.2 (25.1) | 8.4 (25.7) | 11.3 (24.8) | 0.155 |
| Days_post_RRTMean (SD) | 12.0 (19.5) | 18.0 (26.4) | 8.5 (12.9) | <0.001 |
| Sessions Mean (SD) | 12.2 (17.0) | 14.7 (17.7) | 10.8 (16.4) | 0.009 |

ACKD: acute-on-chronic kidney disease; CRS: cadiorenal syndrome; HRS: hepatorenal syndrome; RRT: renal replacement therapy; PD: peritoneal dialysis; CVVHD: continuous hemodialysis; IHD: intermittent hemodialysis; SLED: extended slow-efficiency dialysis.

1. There was 7 missing values in this category.

**3 Sensitive analysis – Hospital Size**

As expected, the stratification of patients according to hospital size yield significant difference in clinical characteristics, disease presentation and outcomes (eTable 3a). In order to minimize individual hospitals bias in the logistic model, we performed a hierarchical mixed-effect model taking individual hospitals as random effect. To explore the potential influence of hospital dichotomization according to number of beds, we performed a further analysis including hospital size in the original model (eTable 3b), and also conducted another hierarchical logistic model including three levels (the same fixed-effects covariates of original multilevel model, with individual hospitals and hospital size as random effects [eTable 3c]). In all above scenarios, after including hospital size in the analysis, no significant difference to the original model assumptions were observed.

Table 3a. Demography and clinical characteristics of patients according to hospital size

|  | Overall (n=170) | Small (n=57) | Medium (n=72) | Large (n=41) | p |
| --- | --- | --- | --- | --- | --- |
| Number of Patients | 17158 | 2281 | 6973 | 7904 |  |
| Age (median [IQR]) | 74.00 [59.00, 83.00] | 76.00 [63.00, 84.00] | 74.00 [60.00, 83.00] | 73.00 [57.00, 82.00] | <0.001 |
| Male gender (%) | 9302 (54.2) | 1236 (54.2) | 3726 (53.4) | 4340 (54.9) | 0.197 |
| ICU admission (%) | 14710 (85.7) | 2109 (92.5) | 6278 (90.0) | 6323 (80.0) | <0.001 |
| Sepsis on admission (%) | 7767 (45.3) | 1060 (46.5) | 3554 (51.0) | 3153 (39.9) | <0.001 |
| Later sepsis (%) | 4598 (26.8) | 625 (27.4) | 1757 (25.2) | 2216 (28.0) | <0.001 |
| Liver disfunction (%) | 334 (1.9) | 35 (1.5) | 134 (1.9) | 165 (2.1) | 0.237 |
| Oliguria (%) | 13007 (75.8) | 1641 (71.9) | 5439 (78.0) | 5927 (75.0) | <0.001 |
| ACKD (%) | 5440 (31.7) | 817 (35.8) | 2177 (31.2) | 2446 (30.9) | <0.001 |
| Community-acquired AKI (%) | 4698 (27.4) | 564 (24.7) | 1982 (28.4) | 2152 (27.2) | 0.002 |
| Mechanical ventilation (%) | 12829 (74.8) | 1778 (77.9) | 5276 (75.7) | 5775 (73.1) | <0.001 |
| Vasopressors (%) | 12122 (70.6) | 1695 (74.3) | 4937 (70.8) | 5490 (69.5) | <0.001 |
| Non-renal organ failure (mean (SD)) | 2.02 (1.23) | 2.09 (1.19) | 1.99 (1.20) | 2.02 (1.27) | 0.007 |
| Charlson score (mean (SD)) | 2.03 (0.88) | 2.05 (0.87) | 2.01 (0.87) | 2.03 (0.88) | 0.157 |
| Mortality (%) | 12288 (71.6) | 1685 (73.9) | 5110 (73.3) | 5493 (69.5) | <0.001 |

ICU: intensive care unit; ACKD: acute-on-chronic kidney disease; AKI: acute kidney injury

Table 3b. Addition of hospital size in the original multilevel logistic regression model

| Fixed effects: |  |  |  |  |
| --- | --- | --- | --- | --- |
|  | Estimate | Std. Error | z value | Pr(>\|z\|) |
| (Intercept) | -2.087.917 | 0.142860 | -14.615 | < 2e-16 *** |
| Age | 0.021289 | 0.001142 | 18.638 | < 2e-16 *** |
| Male Gender | -0.134715 | 0.042130 | -3.198 | 0.00139 ** |
| Sepsis admission | 0.468326 | 0.050007 | 9.365 | < 2e-16 *** |
| Later sepsis | 0.311715 | 0.060240 | 5.175 | 2.28e-07 *** |
| Liver disfunction | 1.563.786 | 0.215294 | 7.263 | 3.77e-13 *** |
| Public hospital | 0.076572 | 0.150009 | 0.510 | 0.60974 |
| ACKD | -0.124711 | 0.046278 | -2.695 | 0.00704 ** |
| ICU admission | -0.393249 | 0.061359 | -6.409 | 1.46e-10 *** |
| Community-acquired AKI | -0.458699 | 0.049108 | -9.341 | < 2e-16 *** |
| Oliguria | 0.382532 | 0.047893 | 7.987 | 1.38e-15 *** |
| Charlson score | 0.054011 | 0.025385 | 2.128 | 0.03336 * |
| Mechanical ventilation | 0.487649 | 0.073453 | 6.639 | 3.16e-11 *** |
| Vasopressors | 0.453457 | 0.069846 | 6.492 | 8.45e-11 *** |
| Non-renal organ failure (per organ) | 0.304700 | 0.033902 | 8.988 | < 2e-16 *** |
| **Hospital size: Small (< 50 beds)** | **Reference** | **-** | **-** | **-** |
| **Medium (51-150 beds)** | **0.169784** | **0.118061** | **1.438** | **0.15040** |
| **Large (> 151 beds)** | **0.123951** | **0.143393** | **0.864** | **0.38736** |
|  |  |  |  |  |
| Signif. codes: 0 ‘***’ 0.001 ‘**’ 0.01 ‘*’ 0.05 ‘.’ 0.1 ‘ ’ 1 |  |  |  |  |
|  |  |  |  |  |
| Random effects: |  |  |  |  |
| Groups Name Variance Std.Dev. |  |  |  |  |
| Hospital (Intercept) 0.1705 0.4129 |  |  |  |  |
| Number of obs: 17158, groups: Hospital, 170 |  |  |  |  |

Table 3c. Three-level hierarchical logistic regression (individual hospitals and hospital size as random-effects)

| Fixed effects: |  |  |  |  |
| --- | --- | --- | --- | --- |
|  | Estimate | Std. Error | z value | Pr(>\|z\|) |
| (Intercept) | -1.973.713 | 0.117509 | -16.796 | < 2e-16 *** |
| Age | 0.021251 | 0.001142 | 18.602 | < 2e-16 *** |
| Male Gender | -0.135124 | 0.042100 | -3.210 | 0.00133 ** |
| Sepsis admission | 0.468686 | 0.049957 | 9.382 | < 2e-16 *** |
| Later sepsis | 0.311767 | 0.060196 | 5.179 | 2.23e-07 *** |
| Liver disfunction | 1.564.324 | 0.214928 | 7.278 | 3.38e-13 *** |
| Public hospital | 0.087947 | 0.135786 | 0.648 | 0.51719 |
| ACKD | -0.125196 | 0.046245 | -2.707 | 0.00678 ** |
| ICU admission | -0.392455 | 0.061293 | -6.403 | 1.52e-10 *** |
| Community-acquired AKI | -0.458023 | 0.049081 | -9.332 | < 2e-16 *** |
| Oliguria | 0.383404 | 0.047862 | 8.011 | 1.14e-15 *** |
| Charlson score | 0.054297 | 0.025363 | 2.141 | 0.03229 * |
| Mechanical ventilation | 0.487100 | 0.073417 | 6.635 | 3.25e-11 *** |
| Vasopressors | 0.452836 | 0.069810 | 6.487 | 8.77e-11 *** |
| Non-renal organ failure (per organ) | 0.304403 | 0.033873 | 8.987 | < 2e-16 *** |
|  |  |  |  |  |
| Signif. codes: 0 ‘***’ 0.001 ‘**’ 0.01 ‘*’ 0.05 ‘.’ 0.1 ‘ ’ 1 |  |  |  |  |
| Random effects: |  |  |  |  |
| Groups Name Variance Std.Dev. |  |  |  |  |
| Hospital (Intercept) 1.717e-01 0.4143472 |  |  |  |  |
| Hospital.Size (Intercept) 2.500e-08 0.0001581 |  |  |  |  |
| Number of obs: 17158, groups: Hospital, 170; Hospital.Size, 3 |  |  |  |  |

**4 Supplementary table**

Table 4. Discharge according to RRT method

|  | Discharge in RRT | Full recovery | Partical recovery | Unknown recovery |
| --- | --- | --- | --- | --- |
| IHD | 739 (28.7%) | 602 (23.4%) | 864 (33.5%) | 373 (14.5%) |
| PIRRT | 279 (17.8%) | 441 (28.1%) | 528 (33.7%) | 319 (20.4%) |
| C-PIRRT | 86 (14.7%) | 208 (35.5%) | 172 (29.4%) | 120 (20.5%) |
| PD | 29 (22.3%) | 46 (35.4%) | 42 (32.3%) | 13 (10.0%) |
| Total | 1133 (23.3%) | 1297 (26.7%) | 1606 (33.0%) | 825 (17.0%) |

IHD, intermittent conventional hemodialysis; PIRRT, prolongated intermittent renal replacement therapy; C-PIRRT, PIRRT, continuous mode; PD: continuous automatized peritoneal dialysis
