# Supplementary material for: A real-world prospective study on dialysis-requiring acute kidney injury
Source: PLoS One. 2022 May 5;17(5):e0267712. doi: 10.1371/journal.pone.0267712 (PMC9071163; doi:10.1371/journal.pone.0267712)
Supplement: S2 File — (DOCX) [file pone.0267712.s002.docx]

**S2 -** Supplementary Figures

**eFigure 1**. Age distribuition across study period. A) Time series with smooth line trend in age distribution and B) blox-plot representation of age distribuition in the 11-year period.

**eFigure 2**. The most frequent clinical and surgical etiologies and conditions associated with Acute Kidney Injury Requiring Dialysis.

**eFigure 3**. Concurrent failing organs in addition to AKI in each of 13 age strata.

**eFigure 4.** Temporal trends in incident causes of severe AKI in the 11-year period

**eFigure 5**. Number of patients per year and crude mortality rates over 11-year period.

**eFigure 6**. Number of concurrent failing organs in addition to AKI in survivors and non-survivors.

**eFigure 7**. Survival curves according to Kaplan-Meier estimates, stratified by number of organ failures

**eFigure 8**. Discharge outcomes according to AKI phenotype.

A)

**
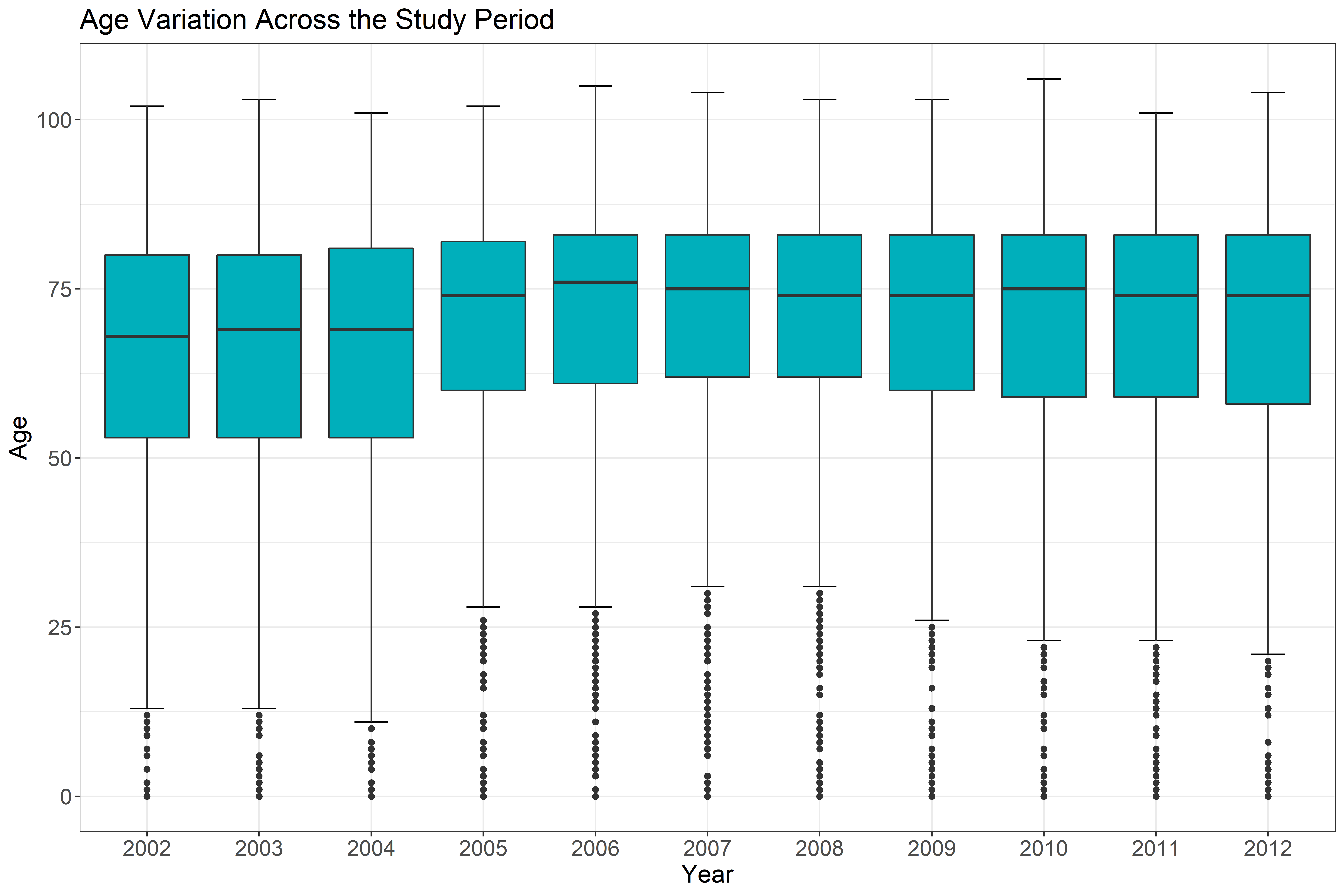

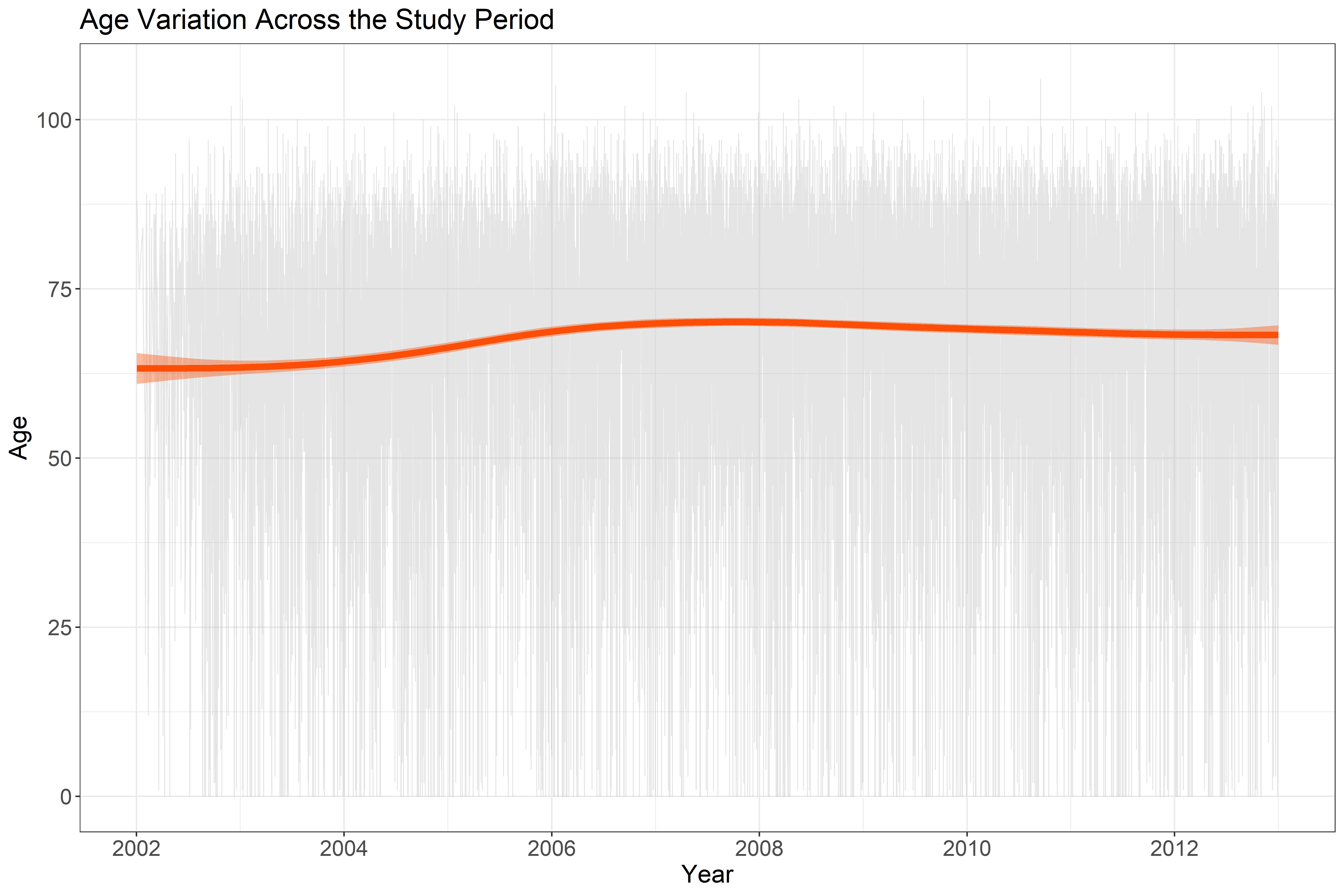
**

B)

**eFigure 1. Age distribution across study period. A) Time series with smooth line trend in age distribution and B) Blox-plot representation of age distribution in the 11-year period.**


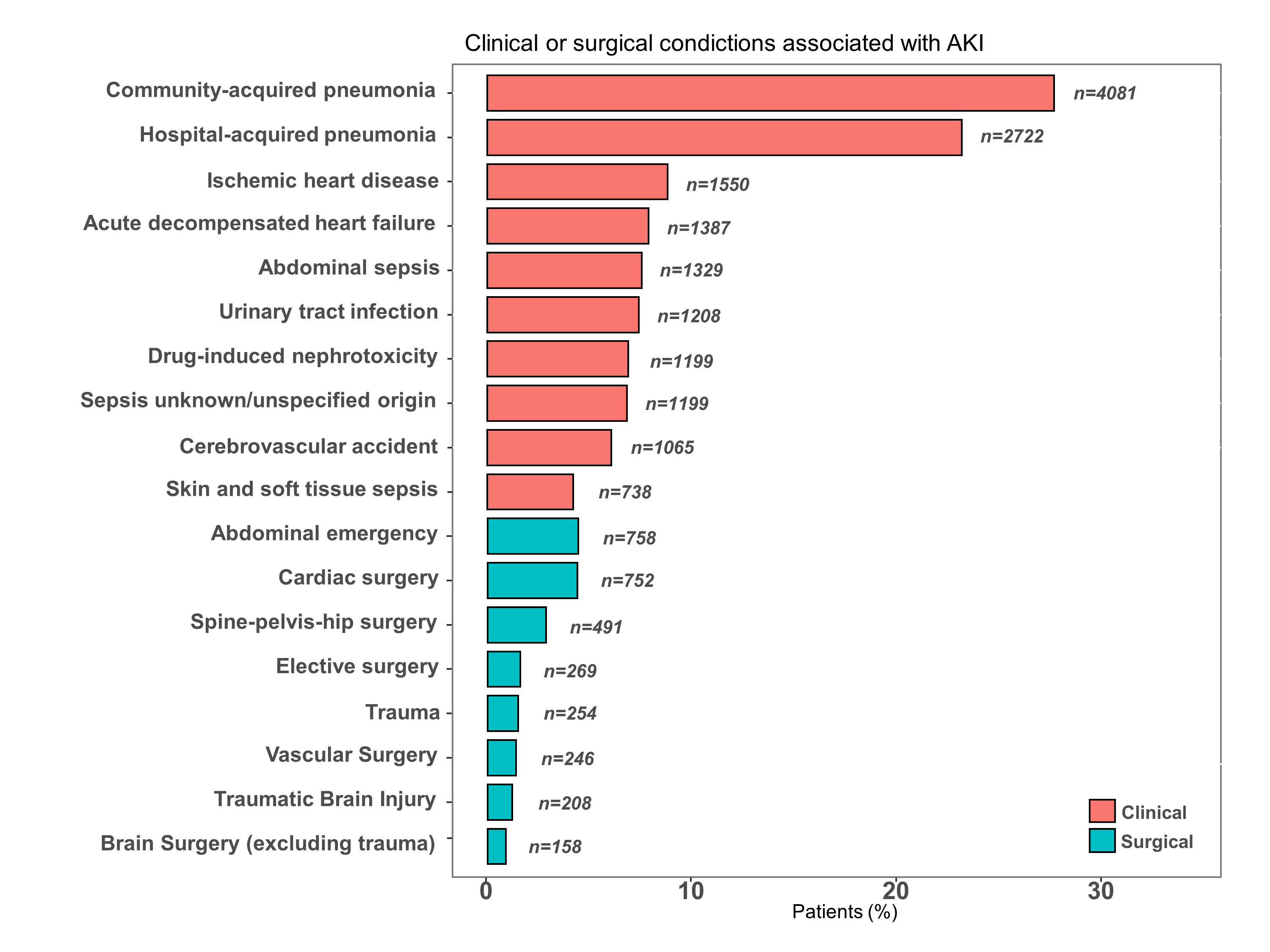


**eFigure 2. The most frequent clinical and surgical etiologies and conditions associated with Acute Kidney Injury Requiring Dialysis**


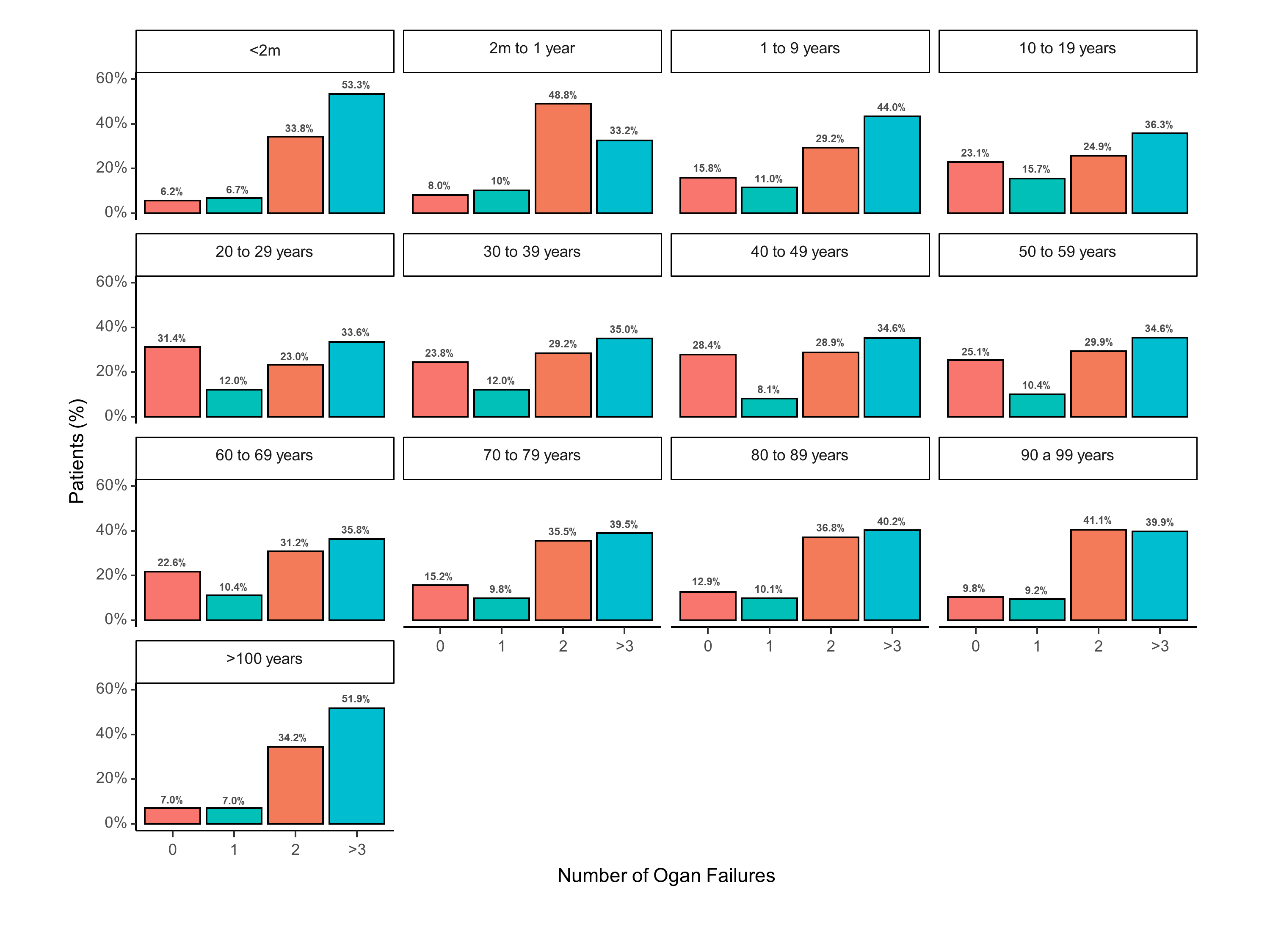


**eFigure 3. Concurrent failing organs in addition to AKI in each of 13 age strata.**

**
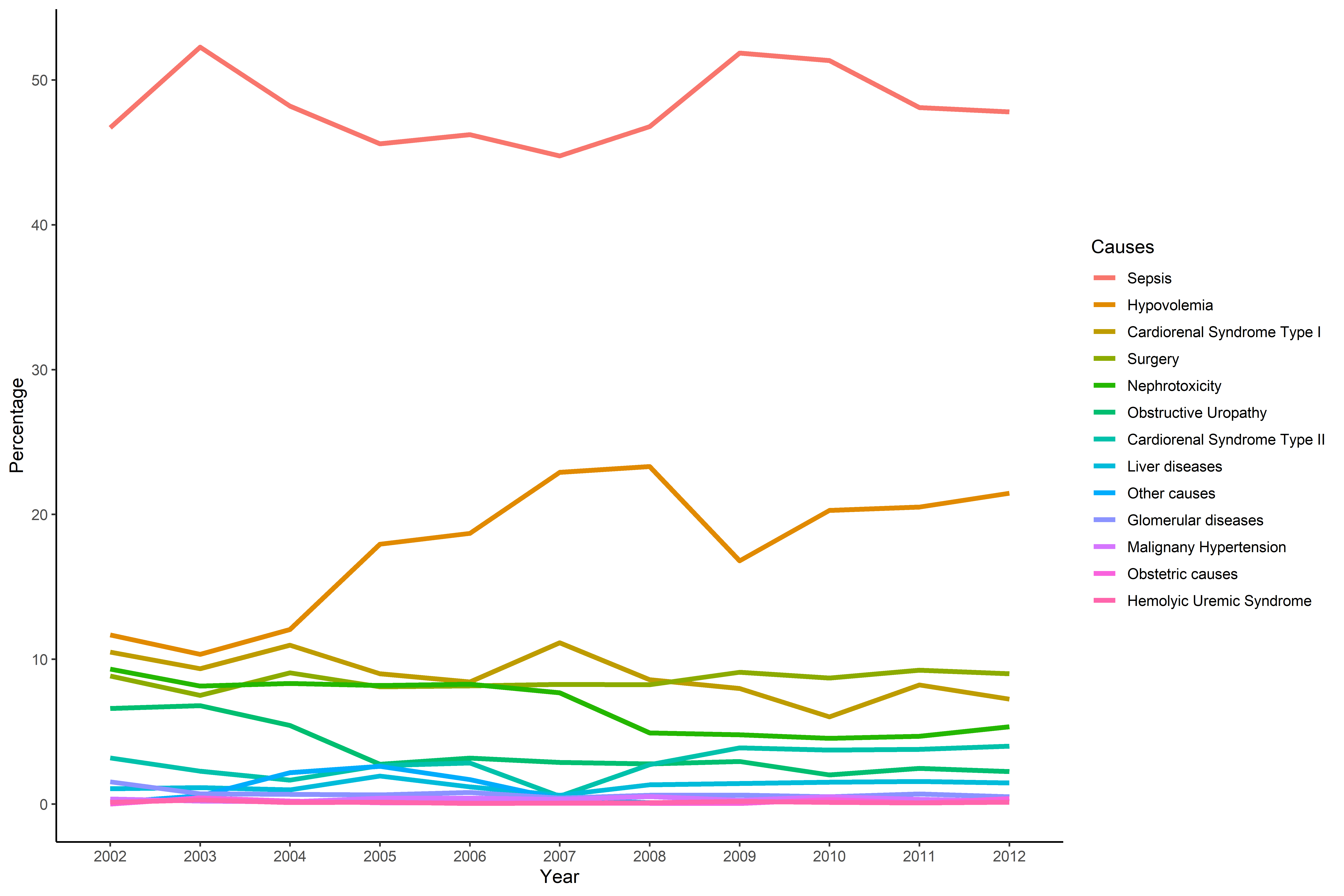
**

**
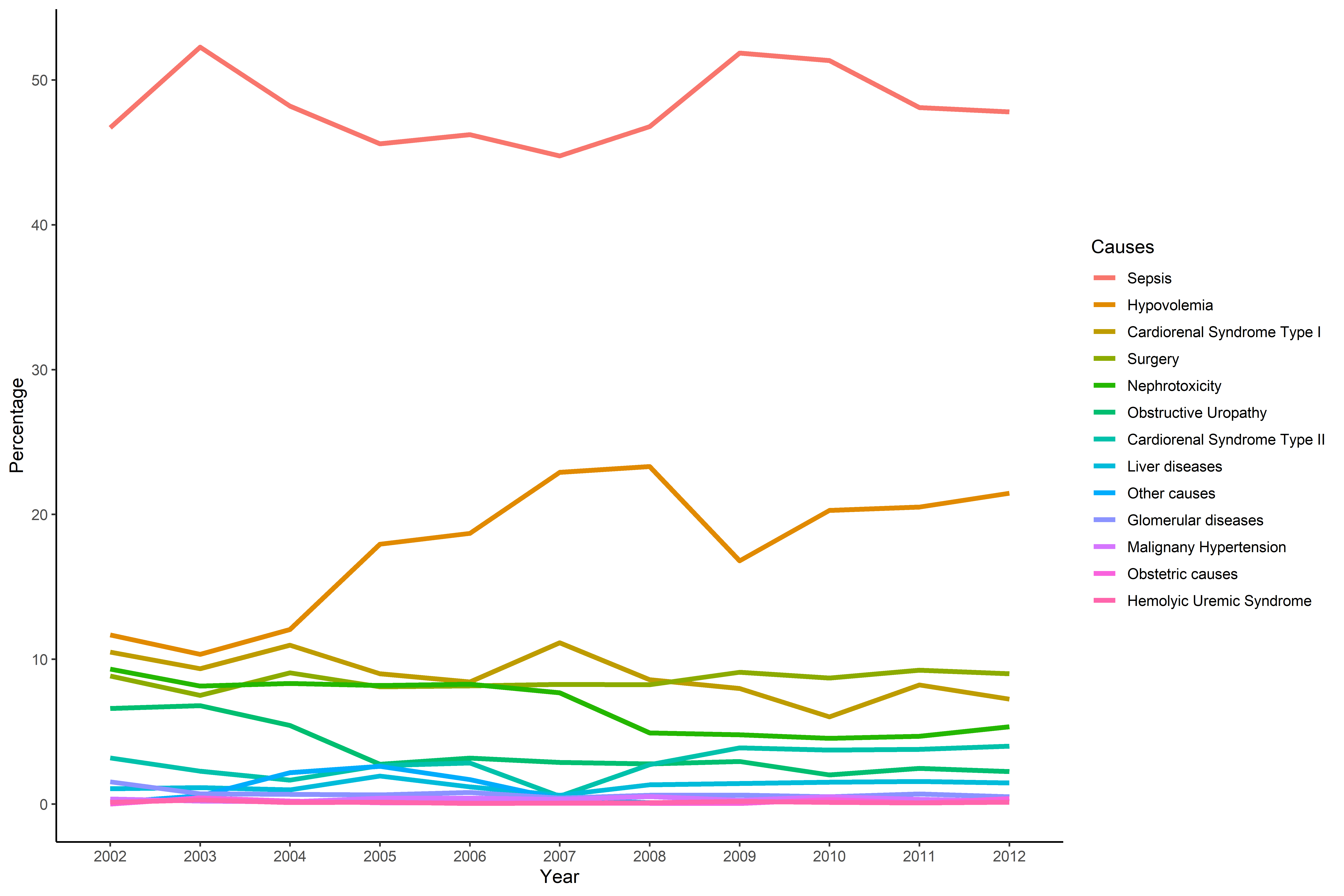
**

**eFigure 4. Temporal trends in incident causes of severe AKI in the 11-year period**

**
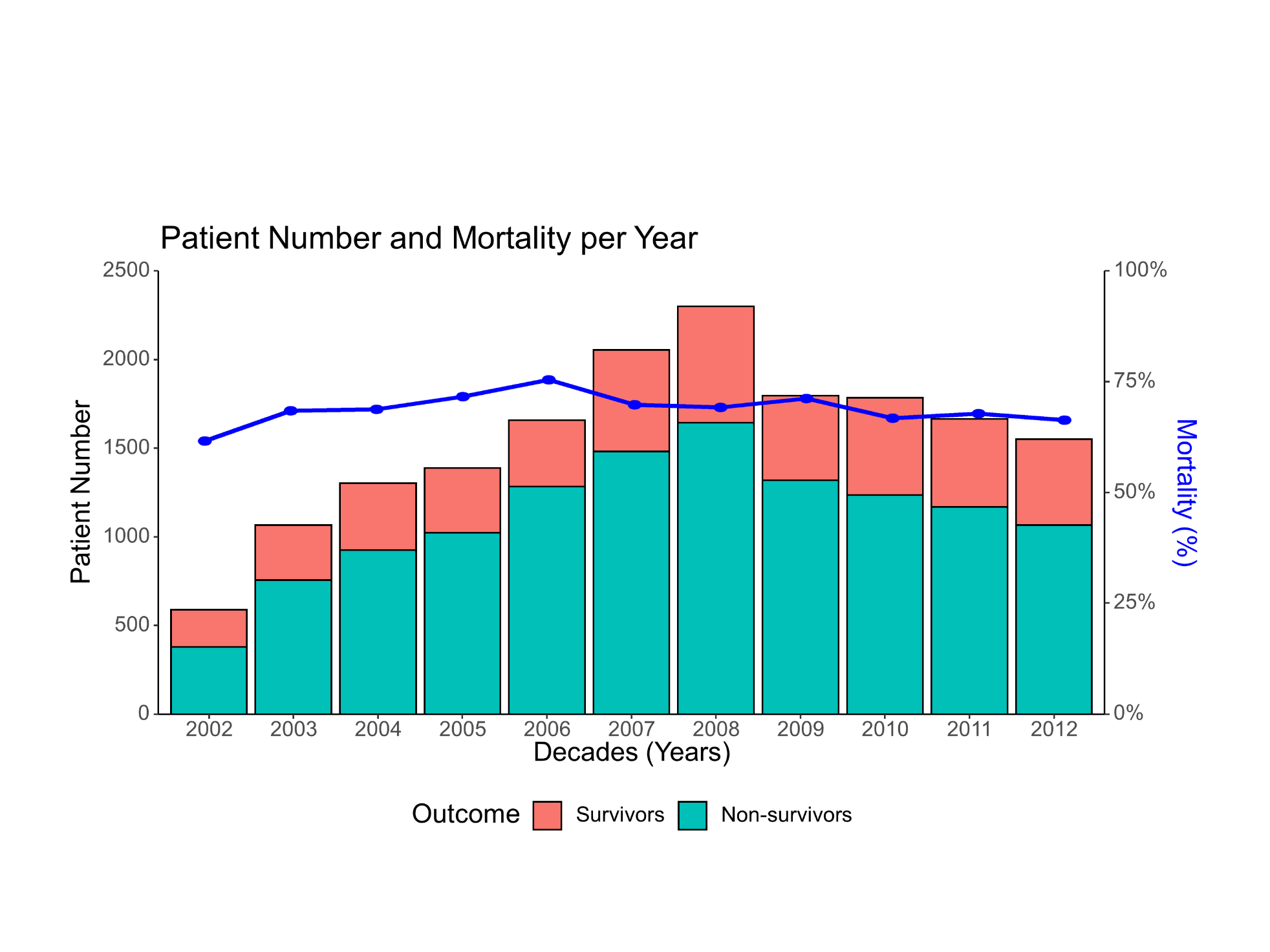
**

**eFigure 5. Number of patients per year and crude mortality rates over 11-year period.**


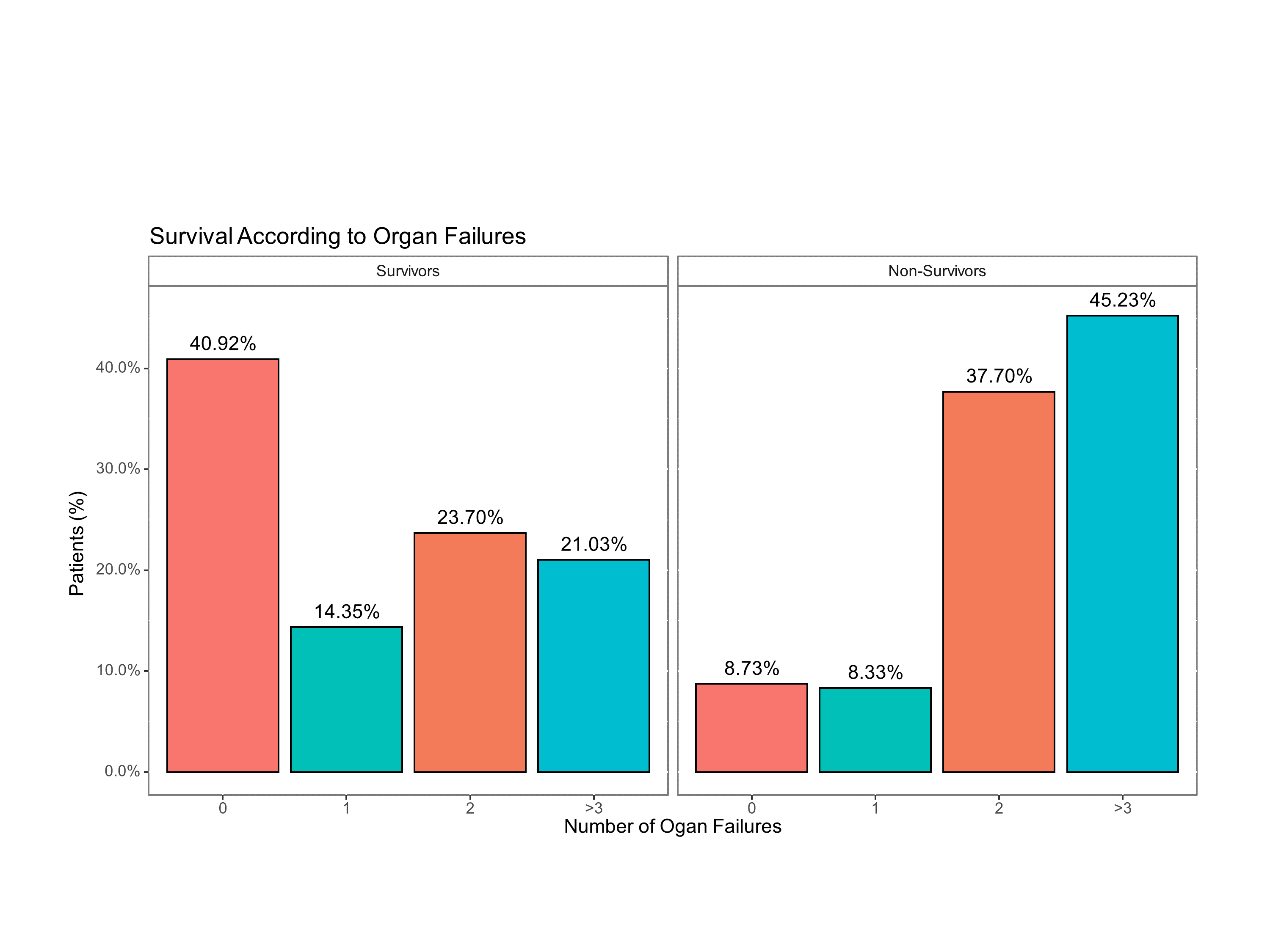


**eFigure 6. Number of concurrent failing organs in addition to AKI in survivors and non-survivors.**

**
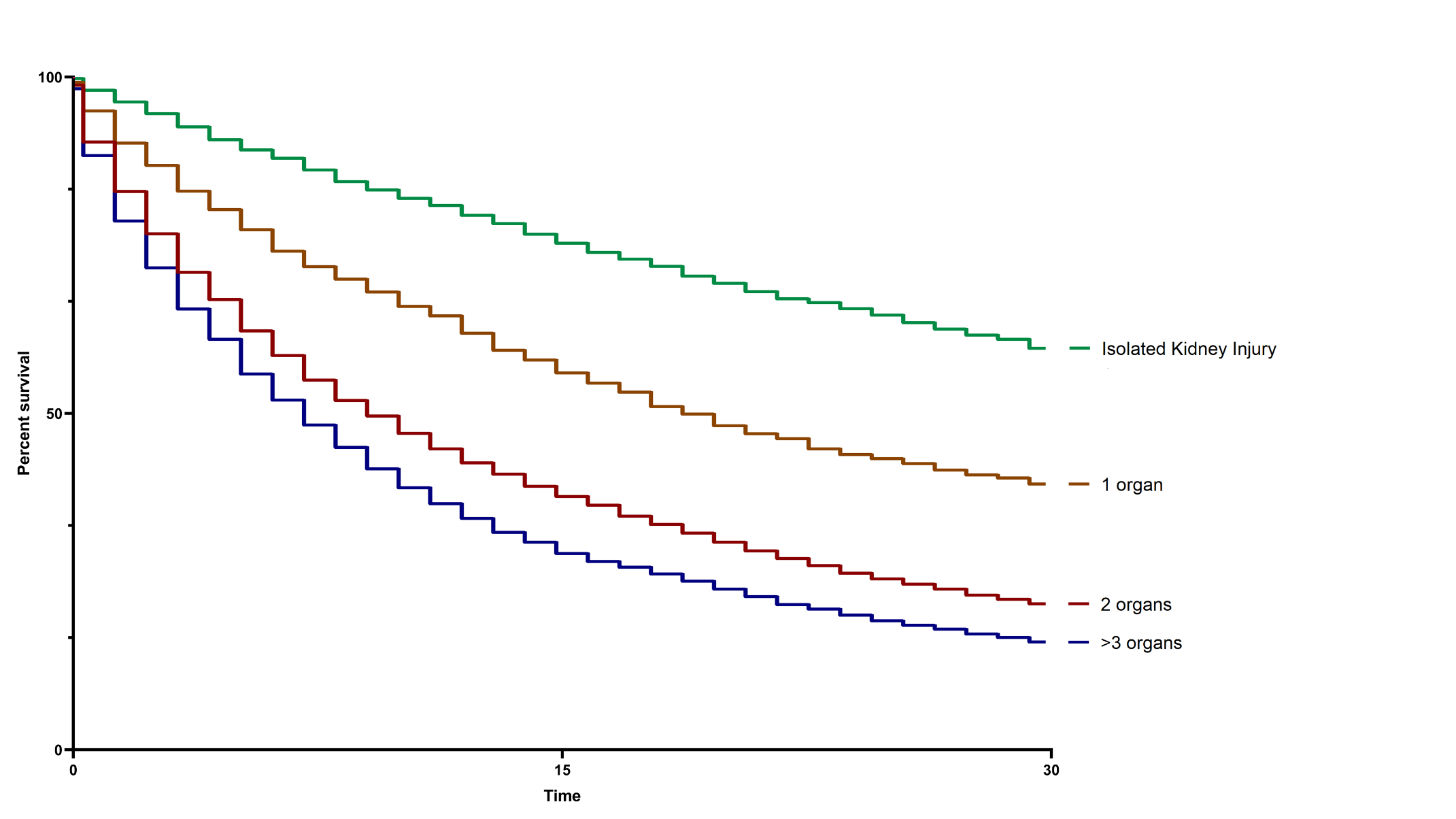
eFigure 7. Kaplan-Meier survival estimates stratified by the number of additional acute organ failures besides acute kidney injury (log-rank test, p<0.0001).**

**
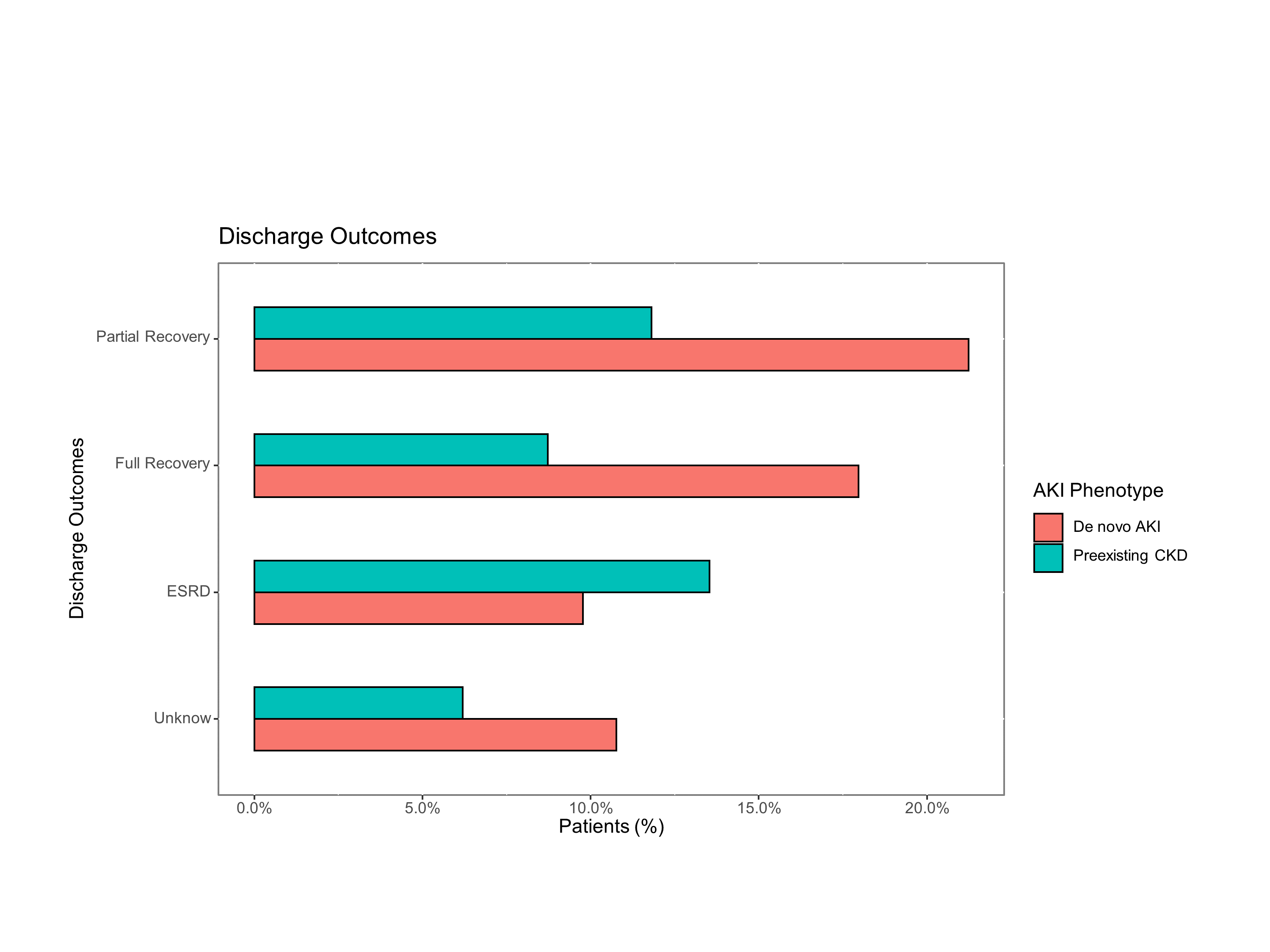
**

**eFigure 8. Discharge outcomes according to AKI phenotype.**
